# Punicalagin Enhances Autophagy Through Sirtuin 1/FoxO3a Axis to Inhibit Intracellular *Mycobacterium Abscessus* Infection

**DOI:** 10.1002/advs.202511734

**Published:** 2025-10-14

**Authors:** Kefan Bi, Bihan Xu, Dan Cao, Kaijin Xu, Ying Zhang

**Affiliations:** ^1^ State Key Laboratory for Diagnosis and Treatment of Infectious Diseases National Clinical Research Center for Infectious Diseases China‐Singapore Belt and Road Joint Laboratory on Infection Research and Drug Development National Medical Center for Infectious Diseases Collaborative Innovation Center for Diagnosis and Treatment of Infectious Diseases The First Affiliated Hospital Zhejiang University School of Medicine Hang Zhou Zhe Jiang 310003 China; ^2^ Yuhang Institute for Collaborative Innovation and Translational Research in Life Sciences and Technology Hang Zhou Zhe Jiang 310003 China; ^3^ Jinan Microecological Biomedicine Shandong Laboratory Jinan Shandong 250022 China

**Keywords:** autophagy, host‐directed therapy, macrophage, *Mycobacterium abscessus*, Punicalagin

## Abstract

*Mycobacterium abscessus* (MAB) is an increasingly recognized rapidly growing nontuberculous mycobacterial pathogen whose infection is particularly challenging to treat due to its antibiotic resistance and persistence, necessitating the exploration of innovative treatment strategies. In this study, it is demonstrated that punicalagin, a polyphenolic compound derived from pomegranate, enhances macrophage antibacterial activity by promoting autophagy rather than exerting direct bactericidal effects. In THP‐1 macrophages, punicalagin at 40 µm reduced intracellular MAB by 47% at 24 h post‐infection. Mechanistically, punicalagin treatment induced an increase LC3‐II/LC3‐I ratio and p62 degradation. It is found that punicalagin promotes autophagy by enhancing mitochondrial stability through upregulating SIRT1 and activating the SIRT1/FoxO3 axis, which in turn inhibits the PI3K/Akt/mTOR pathway. In vivo mouse studies show that punicalagin treatment significantly reduce the MAB burden in the lungs and alleviates the inflammatory cell infiltration in infected lung tissue. The investigation reveals a striking cellular selectivity in its mechanism of action. Punicalagin demonstrates preferential efficacy in interstitial macrophages, while exhibiting little impact on the MAB burden within alveolar macrophages. Transcriptomic analysis of sorted macrophage populations confirms a significant enrichment of autophagy and lysosome‐related pathways specifically in IMs from punicalagin‐treated mice. Taken together, the findings uncover a novel host‐directed therapeutic strategy against MAB infection.

## Graphical abstract

Punicalagin, a pomegranate molecule, helps the body fight *Mycobacterium abscessus*. It preferentially boosts interstitial macrophages in the lung, stabilizing mitochondria and switching on the autophagy via SIRT1/FoxO3a, which lowers bacterial load without directly killing bacteria. The findings highlight a targeted, host‐directed strategy that complements existing treatments and may improve difficult‐to‐treat *Mycobacterium abscessus* infections.

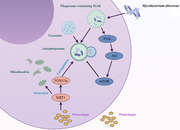

## 1 Introduction

*Mycobacterium abscessus* (MAB) is an emerging rapidly growing non‐tuberculous mycobacterium (NTM) that has shown a global increase in incidence in recent years.^[^ ^1^ ^]^ Historically, the majority of MAB infections are believed to result from opportunistic infections linked to soil and water contamination.^[^ ^2^ ^]^ However, recent research has shown a significant increase in the virulence and transmission capability of MAB, particularly among patients with compromised immune system and respiratory conditions such as cystic fibrosis, chronic obstructive pulmonary disease, and bronchiectasis.^[^ ^3^, ^4^ ^]^ An extensive analysis of 38 686 NTM clinical isolates collected globally between 2004 and 2017 revealed that MAB was most identified in Asia (16%) and Oceania (12%), while its prevalence was lower in South America (5.7%), North America (3.2%), and Europe (2.9%).^[^ ^5^ ^]^ Furthermore, a 15‐year follow‐up study of 1445 patients newly diagnosed with NTM pulmonary disease between July 1997 and December 2013 reported cumulative mortality rates of 11%, 30%, and 50% at 5, 10, and 15 years, respectively, for lung disease associated with MAB infection.^[^ ^6^ ^]^

The treatment of MAB infections poses significant challenges within the clinical realm. Due to its inherent resistance to antibiotics, MAB is typically unresponsive to conventional monotherapy and is also resistant to most anti‐tuberculosis drugs.^[^ ^1^, ^7^, ^8^ ^]^ Current guidelines and expert consensus typically recommend using a combination of three to five antimicrobial agents. The standard treatment for MAB commonly includes macrolides such as clarithromycin or azithromycin, cefoxitin or imipenem, and amikacin. The emergence of drug resistance to the treatments necessitates the development of new therapeutic approaches.^[^ ^3^ ^]^ Besides the conventional antibiotics used in the clinical treatment of MAB infections, recent studies to have shown certain herbal medicines and essential oils to have promising antibacterial effects,^[^ ^9^ ^]^ particularly against the biofilm‐form of MAB, which may have implications for treatment.^[^ ^10^ ^]^ The interaction between mycobacteria and their host is complex and dynamic, influencing the outcome of infections. MAB has evolved multiple strategies to interfere with or evade host innate signaling pathways and phagocytosis and evading reactive oxygen species (ROS). Such events can modulate the host‐pathogen relationship, favoring the survival of mycobacteria within macrophages.^[^ ^9^ ^]^

MAB can grow intracellularly, particularly within macrophages, which are the primary cellular targets during infection.^[^ ^11^, ^12^ ^]^ In the lung, alveolar macrophages (AMs) and interstitial macrophages (IMs) occupy distinct niches and perform complementary, non‐redundant roles during the early stages of mycobacterial infection.^[^ ^14^ ^]^ In murine models of *Mycobacterium tuberculosis*, AMs are the primary host cells supporting productive infection during the early phase following aerosol exposure, productive infection during the first few days after aerosol exposure occurs predominantly within airway resident AMs, and infected AMs subsequently relocate to the interstitium through mechanisms that require ESX‐1 secretion and signaling through the interleukin‐1 receptor.^[^ ^15^ ^]^ In vivo, AMs upregulate an NRF2 dependent antioxidant program that attenuates early bactericidal activity and thereby renders them permissive to infection. In contrast, IMs differ in ontogeny, localization and metabolism and exhibit stronger intrinsic antimicrobial activity that constrains mycobacterial growth.^[^ ^16^ ^]^ Macrophage infection by mycobacteria initiates an inflammatory response through the binding of PAMPs with pattern recognition receptors (PRRs) and NOD‐like receptors (NLRs).^[^ ^17^ ^]^ Specifically, TLR1(Toll‐Like Receptor 1), TLR2(Toll‐Like Receptor 2), and TLR6(Toll‐Like Receptor 6) recognize various mycobacterial components including lipoproteins, proteins, and glycolipids, while TLR3, TLR7, and TLR8 respond to mycobacterial nucleic acids, thereby activating the immune response.^[^ ^18^, ^19^ ^]^ Autophagy is a crucial cellular process that degrades damaged organelles, bacteria, and proteins.^[^ ^18^ ^]^ Autophagy‐related genes (ATGs) facilitate the formation of autophagosomes which fuse with lysosomes to degrade their contents, thus maintaining cellular homeostasis.^[^ ^20^ ^]^ However, mycobacteria have evolved complex mechanisms to evade this host defense. MAB could evade autophagy mediated by macrophages and avoid degradation within the lysosomal compartment.^[^ ^21^, ^22^ ^]^ Notably, MAB produces glycopeptidolipids that inhibit ROS‐mediated apoptosis and suppress the induction of autophagy.^[^ ^21^ ^]^ Some studies suggest that the production of intracellular ROS may promote the growth of MAB, although current research on this topic is limited.^[^ ^23^ ^]^ MAB infection exacerbates mitochondrial oxidative stress, promotes the release of oxidized mitochondrial DNA, enhances IFN‐I secretion, and alters mitochondrial fatty acid metabolism pathways, thereby facilitating its intracellular persistence.^[^ ^9^, ^24^ ^]^

Host‐directed therapy (HDT) has emerged as a vital strategy for enhancing pathogen clearance, particularly in the context of mycobacterial infections that are refractory to conventional antibiotics.^[^ ^25^, ^26^ ^]^ Rather than targeting the microorganism directly, HDT aims to modulate host cellular pathways to strengthen innate immune defenses, limit immunopathology, and promote microbial eradication.^[^ ^25^ ^]^ This approach encompasses a range of interventions designed to enhance phagocytic and autophagic functions, integrate innate and adaptive immunity, mitigate excessive inflammation and tissue damage, reduce apoptosis, and counteract host factors that facilitate mycobacterial persistence.^[^ ^27^, ^28^ ^]^ HDT originated from early immuno‐nutritional approaches, such as vitamin D supplementation, which was found to enhance cathelicidin production and autophagy in macrophages during mycobacterial infection.^[^ ^26^ ^]^ This concept has evolved into a broader therapeutic strategy that enhances antimicrobial immunity or curbs pathological inflammation by targeting host cellular pathways. Recent advances have identified several promising host‐directed therapy (HDT) agents, including mTOR inhibitors such as everolimus,^[^ ^29^ ^]^ immunometabolic modulators(e.g., metformin, statins), and kinase inhibitors (e.g., imatinib),^[^ ^30^, ^31^, ^32^ ^]^ which will enhance macrophage functions such as autophagy, phagolysosome maturation, and mitochondrial homeostasis. Emerging compounds such as V46 simultaneously activate AMPK and TFEB while suppressing Akt‐mTOR signaling, demonstrate potent activity against MAB in preclinical models, offering a compelling proof of concept for pathogen‐specific HDT.^[^ ^33^ ^]^ Together, these developments underscore the strong translational potential of HDT as a complementary strategy to overcome drug resistance and improve outcomes in difficult‐to‐treat mycobacterial infections. Despite these advances in HDT, agents with demonstrated activity against MAB remain scarce. Pomegranate is widely recognized as a functional food and nutraceutical due to its rich polyphenolic content, including punicalagin, gallic acid, and ellagic acid.^[^ ^34^ ^]^ These compounds have been shown to enhance mitochondrial function and mitigate oxidative stress and inflammation, thereby exhibiting potent in vitro antioxidant activity.^[^ ^35^ ^]^ Punicalagin, the principal bioactive constituent in pomegranate extract and a hydrolyzable ellagitannin. It exhibits diverse biological activities, including anti‐apoptotic, anti‐proliferative, antimicrobial, antiviral, and anti‐tumor effects.^[^ ^36^, ^37^, ^38^ ^]^ Punicalagin has demonstrated in vivo efficacy in infection‐relevant settings. As a Staphylococcus aureus virulence inhibitor that targets Sortase A, punicalagin reduced disease burden in a murine pneumonia model and showed additive benefit with cefotaxime. In the lung, punicalagin protected mice from lipopolysaccharide induced acute lung injury when administered intraperitoneally at 10 to 40 mg kg^−1^, supporting pulmonary relevance and tolerability of systemic dosing.^[^ ^39^, ^40^ ^]^ Ellagitannins that include punicalagin are chemically stable under acidic conditions, and their aqueous solubility facilitates preparation for in vitro assays and parenteral dosing. Although punicalagin has been reported to exhibit in vitro bactericidal activity against mycobacteria, its minimum inhibitory concentration (MIC) is high, ranging from 600–1200 µg mL^−1^, which may limit its direct therapeutic application.^[^ ^41^, ^42^ ^]^ However, its effect on Mycobacteria within host macrophages has not been described. This study aims to investigate whether punicalagin can inhibit MAB in macrophages and its potential as a HDT agent.

## 2 Results

### 2.1 MAB Infection Alters Transcriptomic Profile and Induces Mitochondrial Damage in THP‐1 Macrophages

**Figure 1:**
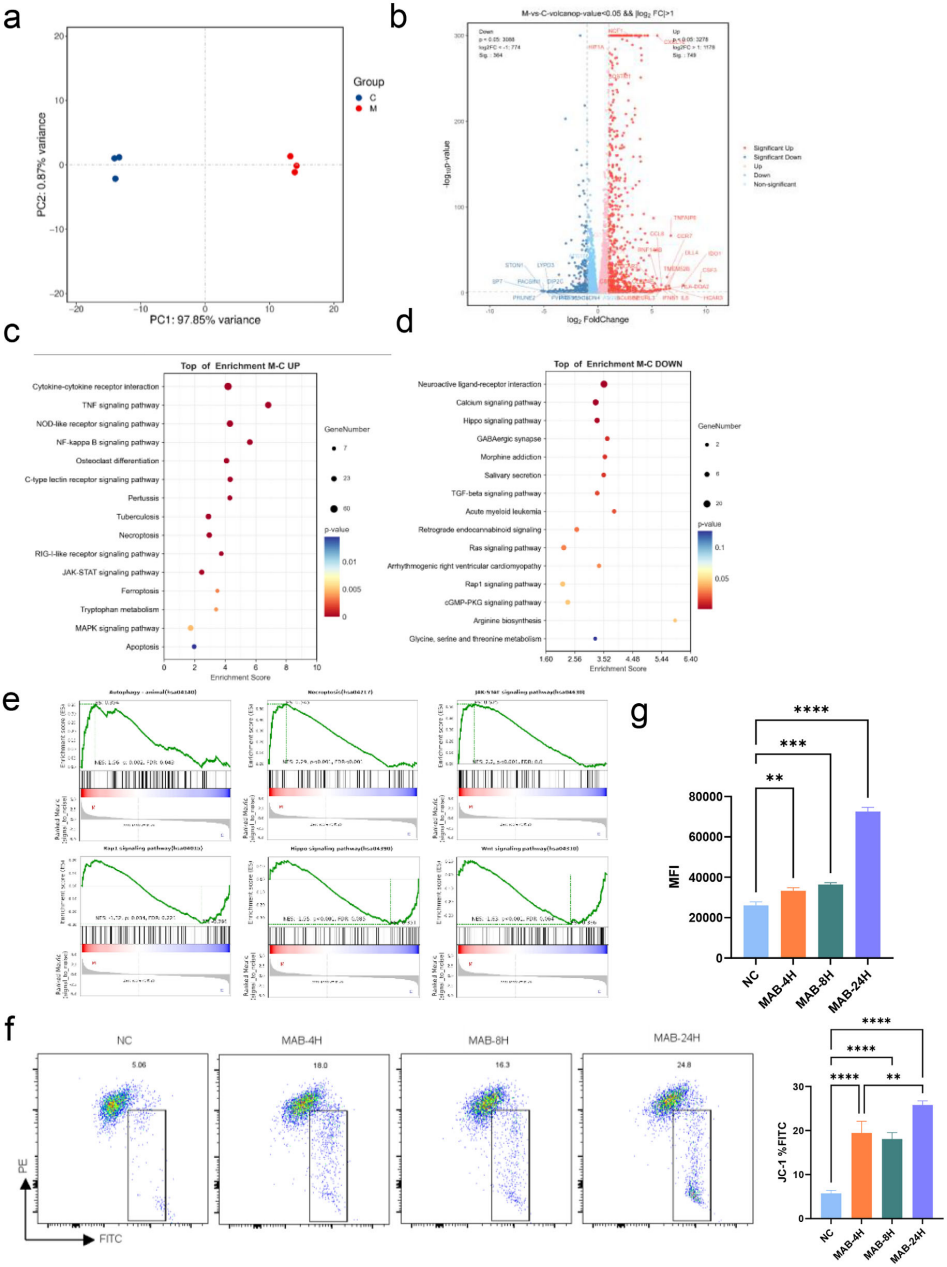
MAB induced mitochondrial damage in macrophages. a) Principal component analysis (PCA). b) Volcano plot of differentially expressed genes. c) KEGG pathway enrichment analysis (upregulation) of the MAB‐infected THP1 transcriptome. d) KEGG pathway enrichment analysis (downregulation) of the MAB‐infected THP1 transcriptome. e) GSEA analysis. f) Δψm was evaluated by flow cytometric analysis of JC‐1 mitochondrial membrane potential dye. g) DCFH‐DA assays. Statistical significance is indicated as *p < 0.05; **p < 0.01; ***p < 0.001; ****p < 0.0001; ns denotes a non‐significant difference. n = 3.

To investigate the differential gene expression in human macrophages infected by MAB, THP‐1 macrophages were infected with the ATCC type strain 19 977 at a multiplicity of infection (MOI) of 10. THP‐1 macrophages were infected with MAB for 4 h, followed by the removal of extracellular bacteria and further incubation for 24 h prior to harvesting for transcriptome sequencing. Comparative transcriptome profiling identified significant differential gene expression in MAB‐infected macrophages relative to uninfected THP‐1 controls (**Figure** **1**a). Transcriptome analysis of MAB‐infected macrophages revealed altered expression of genes governing oxidative stress and autophagy. Upregulation of HIF1A (hypoxia‐inducible factor 1‐alpha) and NCF1 (NADPH oxidase subunit) correlated with heightened intracellular oxidative stress, while downregulation of autophagy regulators ATG16L1 and ATG9 impaired autophagosome formation. This dysregulation enhanced ROS production coupled with suppressed autophagy, suggests a pathogenic strategy by MAB to evade lysosomal degradation and persist within macrophages (Figure 1b). KEGG pathway analysis demonstrated significant activation of TNF and NF‐κB signaling pathways, concurrent with inhibition of Hippo signaling, calcium signaling, and arginine biosynthesis (Figure 1c,d). Notably, GSEA (Gene Set Enrichment Analysis) further corroborated extensive alterations in autophagy‐associated pathways and dysregulation of HIF‐1, Rap1, Wnt, and necroptosis signaling cascades (Figure 1e). Given prior evidence linking HIF‐1, Wnt, and Hippo pathway dysregulation to mitochondrial dysfunction, it was hypothesized that MAB infection disrupts mitochondrial function and redox homeostasis. JC‐1 staining demonstrated a time‐dependent reduction in mitochondrial membrane potential in MAB‐infected cells were assessed at 0, 4, and 24 h post‐infection (hpi) compared to uninfected controls, which demonstrated a reduction in mitochondrial membrane potential induced by MAB infection over time (Figure 1f). Concurrently, DCFH‐DA assays demonstrated a time‐dependent increase in intracellular reactive oxygen species (ROS) levels during the infection (Figure 1g), confirming that mitochondrial dysfunction and ROS production are hallmark features of MAB infection.

### 2.2 Punicalagin Activates Autophagy and Suppresses Intracellular MAB Infection

**Figure 2:**
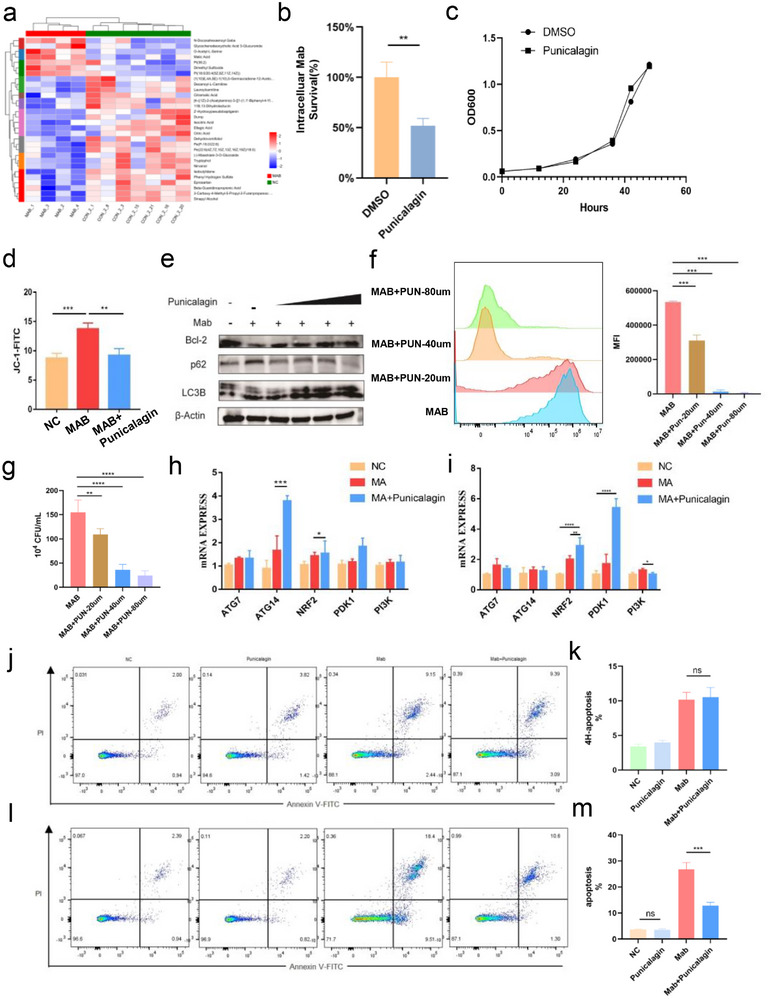
Effects of punicalagin on activation of autophagy in MAB‐infected THP‐1 cells. a) Comparative analysis of peripheral blood metabolomic profiles between *Mycobacterium abscessus*‐infected patients (MAB group) and healthy controls (NC group). b) CFU data at the indicated time post‐infection of MAB‐infected THP‐1 cells. c) Bacterial growth curves in 7H9 culture medium. d) Δψm was evaluated by flow cytometry analysis of JC‐1. e) Representative WB images of autophagy markers in MAB‐infected THP‐1 cells treated with punicalagin. f) Fluorescence intensity of intracellular MAB‐GFP. g) Intracellular MAB culture was treated with punicalagin followed by CFU count. h) The mRNA expression levels of genes involved in autophagy at 24 h post‐infection with MAB. i) mRNA expression levels of genes involved in autophagy 4 h post‐infection. j) THP‐1 apoptosis assay (4 h post‐infection). k) Quantification of apoptosis (4 h post‐infection). l) THP‐1 apoptosis assay (24 h post‐infection). m) Quantification of apoptosis (24 h post‐infection). Statistical significance is indicated as *p < 0.05; **p < 0.01; ***p < 0.001; ****p < 0.0001; ns denotes a non‐significant difference. n = 3.

Untargeted metabolomics analysis was performed on peripheral blood plasma from healthy controls and MAB‐infected patients, which identified numerous differential metabolites (**Figure** **2**a). Ellagic acid is primarily derived from the metabolism of ellagitannins. Based on prior extensive drug screening by our research group, we identified punicalagin as a compound that significantly enhances the inhibition of intracellular MAB (Figure 2b). Punicalagin, the predominant ellagitannin component in pomegranate (*Punica granatum*), serves as a key pro‐drug precursor of ellagic acid and exhibits high stability, treatment of THP‐1 cells with punicalagin followed by 24‐h co‐cultured resulted in the detection of ellagic acid in the culture supernatant via mass spectrometry, indicating that punicalagin undergoes metabolic conversion to ellagic acid during cellular cultivation(Figure S1, Supporting Information).^[^ ^43^ ^]^ Given the physicochemical properties of punicalagin and the metabolic profile observed in MAB‐infected patients, punicalagin is proposed as a promising HDT candidate against MAB, warranting further validation of its intracellular mycobactericidal mechanisms and efficacy. Correspondingly, the direct effect of punicalagin on the growth of MAB in 7H9 liquid culture was assessed, and the results showed that punicalagin had no significant effect on the growth of MAB when applied directly to the bacteria (Figure 2c). We determined the minimum inhibitory concentration (MIC) of punicalagin against MAB using standard broth microdilution methods. Our results show that the MIC of punicalagin against MAB is 1024 µg mL^−1^, while showing no significant inhibitory effect on the growth of *Mycobacterium tuberculosis (Mtb)* (Figure S2, Supporting Information). To assess the mitochondrial protective effects of punicalagin, we measured mitochondrial membrane potential using JC‐1 staining in MAB‐infected THP‐1 macrophages. Punicalagin treatment was found to restore mitochondrial membrane potential in infected macrophages compared to untreated controls (Figure 2d). The results indicate that punicalagin enhances the antibacterial activity of macrophages against MAB while preserving mitochondrial stability.

Given the capacity of punicalagin to enhance macrophage antibacterial activity while preserving mitochondrial membrane potential stability, its concentration‐dependent therapeutic efficacy and the mechanistic interplay with autophagy were subsequently investigated. Building upon the previous transcriptomic findings (Figure 1c,d). It was hypothesized that punicalagin‐mediated bacterial clearance may mediate this effect through restoration of autophagic flux. To evaluate this, THP‐1 macrophages infected with MAB were treated with escalating concentrations of punicalagin (0, 20, 40, 80 µm), followed by quantitative analysis of autophagy and apoptosis markers. Western blotting revealed an increase in the LC3‐II/LC3‐I ratio, concurrent with dose‐dependent p62 degradation, confirming autophagic flux activation specifically in punicalagin‐treated cells (Figure 2e). Flow cytometry was also used to evaluate the survival of GFP‐labeled MAB within THP‐1 cells under varying concentrations of punicalagin treatment. This approach allowed us to assess the inhibitory effect of punicalagin on intracellular MAB infection (Figure 2f), observed a dose‐dependent reduction in the intracellular burden of MAB with increasing concentrations of punicalagin, indicating a significant inhibition of bacterial survival within host cells. Concurrently, the intracellular MAB in THP‐1 cells was quantified to assess the efficacy of punicalagin in reducing bacterial levels within host cells (Figure 2g). A punicalagin concentration of 40 µM for subsequent treatments because treatment with 80 µm punicalagin induced elevated apoptosis rates (Figure S3, Supporting Information). The results indicated that punicalagin (40 µM) could upregulate the autophagy‐related protein ATG14 at 24 h post‐infection compared to the MAB‐infected THP‐1 without punicalagin treatment (Figure 2h,i). The results showed no significant difference in apoptosis levels between the MAB infection and punicalagin groups at 4 h post‐infection (Figure 2j,k), while at 24 h, the apoptosis levels in the punicalagin group were lower than in the MAB infection group (Figure 2l,m). To discern the primary active agent, we investigated the potential contribution of ellagic acid, the hydrolytic metabolite of punicalagin. Although mass spectrometry confirmed the partial conversion of punicalagin to ellagic acid during cellular incubation (Figure S1, Supporting Information), punicalagin remained the predominant molecular species. This is consistent with its known stability in sterile culture conditions, where rapid enzymatic hydrolysis does not occur. Direct comparative experiments showed that at an equimolar concentration (40 µm), punicalagin was more effective at reducing the intracellular MAB burden and exhibited significantly lower cytotoxicity than ellagic acid (Figure S4, Supporting Information). Therefore, these results indicate that the potent host‐directed therapeutic effects observed in our study are primarily attributable to punicalagin. Next, an examined whether punicalagin modulates the expression of autophagy‐related genes at distinct post‐infection time points in both MAB‐infected and punicalagin‐treated MAB‐infected groups. These results revealed that punicalagin mitigates MAB‐induced mitochondrial dysfunction and oxidative stress, enhancing macrophage antibacterial activity via autophagy activation. Its concentration‐dependent efficacy highlights 40 µm as the optimal dose, effectively reducing the intracellular MAB burden while preserving mitochondrial stability and minimizing cell apoptosis.

### 2.3 Punicalagin Induces Autophagy and Enhances Intracellular Clearance of MAB via mTOR and MAPK/ERK Signaling Pathways

**Figure 3:**
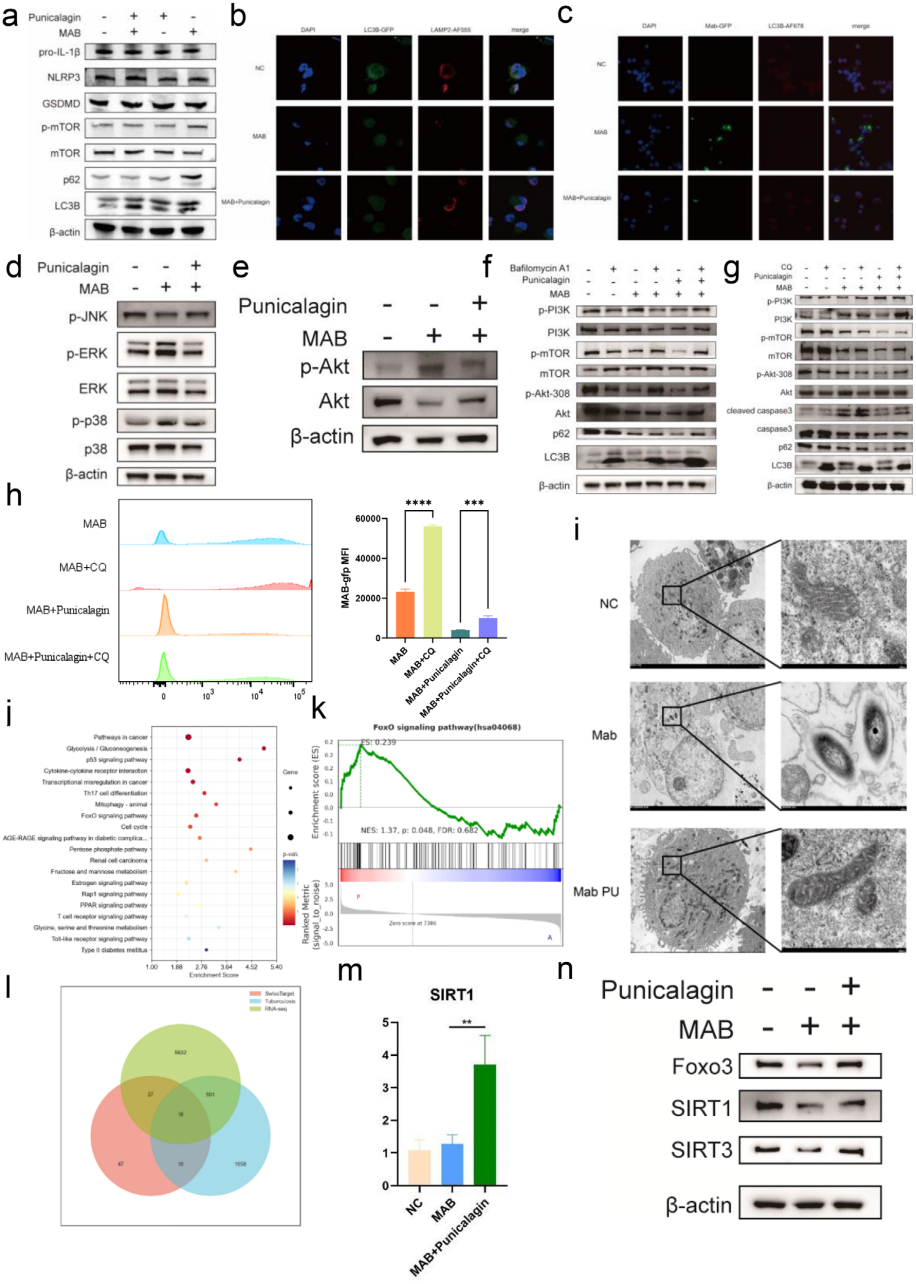
Punicalagin suppresses Akt/mTOR signaling and activates SIRT1 in MAB‐infected THP‐1 cells. a) Representative WB images of autophagy markers in MAB‐infected THP‐1 cells with punicalagin (PUN) treatment. b) Co‐localization of LAMP2 with LC3B. c) Co‐localization of MAB‐GFP with LC3B. d) MAPK representative WB images and Akt representative WB images. e) Akt representative WB images representative WB images. f) Representative WB images upon Bafilomycin A1 treatment. g) Representative WB images upon CQ treatment. h) Fluorescence intensity of intracellular MAB‐GFP with or without CQ. i) Representative transmission electron microscopy images of mitochondria in THP‐1 cells infected with MAB and in MAB‐infected THP‐1 cells treated with Punicalagin. j) KEGG pathway enrichment analysis of the PUN‐treated MAB‐infected and MAB‐infected THP1 transcriptome. k) FoxO GSEA enrichment analysis. l) Venn diagram displaying the important genes. m) SIRT1 mRNA expression. n) SIRT1, SIRT3, and FoxO3a representative WB images. Statistical significance is indicated as *p < 0.05; **p < 0.01; ***p < 0.001; ****p < 0.0001; ns denotes a non‐significant difference. n = 3.

To further investigate the mechanisms underlying punicalagin's therapeutic effects against MAB infection, a series of experiments to examine its influence on cellular signaling pathways and autophagy‐related processes. The results demonstrated that punicalagin treatment had no significant effect on pyroptosis‐related proteins (**Figure** **3**a). Notably, it was found that punicalagin treatment significantly inhibited the activation of mTOR, a key negative regulator of autophagy (Figure 3a).^[^ ^44^ ^]^ Immunofluorescence studies revealed that punicalagin treatment promoted autophagy process in MAB‐infected cells, as evidenced by increased colocalization of LC3B and LAMP2, which indicates enhanced fusion between autophagosomes and lysosomes (Figure 3b). Furthermore, punicalagin‐treated THP‐1 cells exhibited a significant reduction in intracellular bacterial load, suggesting that the activation of autophagy contributes to the clearance of MAB (Figure 3c).

In addition to its effects on autophagy, we found that punicalagin inhibited the activation of MAPK/ERK signaling pathway in MAB‐infected cells, while the p‐P38 and p‐Erk in the punicalagin‐treated MAB‐infected cells were significantly downregulated (Figure 3d). Given that MAPK/ERK signaling is known to negatively regulate autophagy, its inhibition by punicalagin may further promote the enhancement of autophagic processes.^[^ ^44^, ^45^ ^]^ By suppressing MAPK/ERK activation, punicalagin potentially created a more favorable environment for autophagy‐mediated bacterial clearance, thereby contributing to the control of MAB infection (Figure 3d). Furthermore, we found that punicalagin treatment inhibited the phosphorylation of AKT in MAB‐infected cells (Figure 3e). AKT phosphorylation plays a pivotal role in regulating key cellular processes, including cell survival, proliferation, and autophagy, and its inhibition by punicalagin may alleviate AKT‐mediated suppression of autophagy, thereby promoting autophagic process and enhancing bacterial clearance. When combined with the suppression of MAPK/ERK signaling, this dual inhibition suggests that punicalagin facilitates autophagy through the coordinated modulation of multiple signaling pathways. This multifaceted mechanism contributes to its therapeutic efficacy against MAB infection. To further validate that the anti‐MAB activity of punicalagin was mediated by the enhancement of autophagic flux, pharmacological inhibitors Bafilomycin A1 (BafA1) and Chloroquine (CQ) to disrupt distinct stages of the autophagy. Treatment with BafA1 led to an accumulation of both LC3‐II and p62, reflecting a blockade of autophagic degradation, when macrophages were co‐treated with punicalagin and BafA1, a more substantial accumulation of LC3‐II was observed compared to cells treated with either punicalagin or BafA1 alone. Furthermore, the punicalagin‐induced degradation of p62 was reversed by BafA1 co‐treatment, with p62 levels remaining elevated. Meanwhile, we observed that the suppression of PI3K/Akt/mTOR pathway activation by punicalagin alone was markedly reversed by BafA1, indicating that punicalagin impairs macrophage autophagic function, thereby promoting the growth of MAB (Figure 3f). Chloroquine (CQ) was found to block punicalagin‐induced autophagy by inhibiting autophagosome‐lysosome fusion, thereby suppressing autophagic flux, CQ alone caused LC3‐II and p62 accumulation, indicating late‐stage autophagy inhibition. Co‐treatment with punicalagin and CQ led to a greater increase in LC3‐II than either treatment alone, supporting that punicalagin promotes autophagosome formation, which accumulates when degradation is blocked by CQ, we also noted a significant upregulation of cleaved caspase‐3 protein following the addition of CQ, indicating an enhancement of apoptosis levels (Figure 3g). We also found that CQ significantly reduced punicalagin‐mediated MAB clearance in THP‐1(Figure 3h), highlighting the necessity of autophagosome‐lysosome fusion for bacterial degradation. These results indicate that punicalagin primarily eliminates intracellular MAB infection by promoting macrophage autophagy. Building on our earlier findings that punicalagin rescued mitochondrial dysfunction in MAB‐infected macrophages, as evidenced by restored membrane potential (Figure 2d), we next investigated its effects on ultrastructural preservation of mitochondria. Transmission electron microscopy revealed that punicalagin treatment ameliorated MAB‐induced mitochondrial cristae disorganization and outer membrane rupture, maintaining structural integrity comparable to that of uninfected controls. This structural preservation is mechanistically aligned with punicalagin's ability to stabilize bioenergetic homeostasis, as structurally intact mitochondria are essential for sustaining ATP synthesis and regulating redox balance (Figure 3i).

To elucidate the mechanisms by which punicalagin promotes the inhibition of mycobacterial growth within macrophages, cells from both the punicalagin‐treated MAB‐infected group and MAB‐infected group were subjected to RNA‐seq analysis. A total of 1762 upregulated genes and 854 downregulated genes were detected in the punicalagin‐treated MAB‐infected group compared to MAB‐infected groups. We conducted KEGG enrichment analysis on differentially expressed genes. Transcriptome profiling revealed significant upregulation of several critical pathways in punicalagin‐treated MAB‐infected THP‐1 cells compared to untreated controls, including the p53 signaling pathway, cytokine‐cytokine receptor interaction, FoxO (transcription factor forkhead box protein O) signaling pathway, Rap1 signaling pathway, and PPAR signaling pathway (*p* < 0.05). Particularly noteworthy was the concurrent activation of the FoxO pathway, a known regulator of autophagy initiation and lysosomal biogenesis,^[^ ^46^ ^]^ which aligns with our hypothesis that punicalagin may exert its anti‐mycobacterial effects through enhanced autophagic clearance (Figure 3j). GSEA revealed a significant positive correlation with the FoxO signaling pathway in the punicalagin‐treated MAB‐infected group compared to the MAB‐infected group (Figure 3k). Integrated bioinformatics analysis by intersecting three datasets from GeneCards, Swiss Target, and the RNA‐seq data identified 16 core differentially expressed genes, including *SIRT1, NFKB1, GPR35, GSK3B, MAP3K8, SRC, ERBB2, LYN, PTGS2, FYN, NQO1, CES2, PTK2, INSR, MIF, SNCA*, which are the common targets in punicalagin‐treated MAB‐infected group (Figure 3l). One such candidate target, Sirtuin 1 (SIRT1), a deacetylase playing a critical role in various cellular functions including regulation of ROS levels and ferroptosis, was further investigated. qRT‐PCR revealed upregulation of Sirt1 expression in macrophages infected with MAB upon punicalagin treatment compared to MAB‐infected macrophages (Figure 3m). The WB result showed that punicalagin treatment upregulated the protein expression levels of SIRT1, SIRT3, FoxO3 in THP‐1 cells infected with MAB (Figure 3n). We also conducted proteomic profiling of THP‐1 cells infected with MAB, with or without punicalagin treatment, identifying 260 upregulated and 216 downregulated proteins (Figure S5a,b, Supporting Information). From the significantly upregulated proteins (RP105/MD‐1, VDR, TLR2, MyD88, ATP6AP1, RAB32) (Figure S5c, Supporting Information), we selected key host defense‐related signaling molecules for molecular docking analysis. We retrieved the corresponding crystal structures from the RCSB PDB database (PDB IDs: 5V39, 6FF8, 1MIF, 4GUM, 2Z7X, 4DOM, 1KKQ, 1IKN, 2JKK, 3B2D). Our docking analysis revealed favorable binding affinities between punicalagin and several host defense proteins. The calculated binding affinities (in kcal/mol) were as follows: 1IKN: −7.5, 1MIF: −5.9, 2JKK: −9.2, 2Z7X: −6.9, 3B2D: −10.0, 4DOM: −9.1, 4GUM: −12.9, 6FF8: −9.2. Given that negative binding energies typically indicate thermodynamically favorable interactions, these results suggest that punicalagin may bind with relatively high affinity to key immune regulators such as Macrophage Migration Inhibitory Factor (MIF, PDB ID: 4GUM, Figure S6a, Supporting Information), Myeloid Differentiation Primary Response 88 (MyD88, PDB ID: 4DOM, Figure S6b, Supporting Information), the RP105/MD‐1/TLR2 complex (PDB ID: 3B2D, Figure S6c, Supporting Information), Protein Tyrosine Kinase 2 (PTK2, PDB ID: 2JKK, Figure S6d, Supporting Information), and Toll‐Like Receptor 2 (TLR2, PDB ID: 2Z7X, Figure S6e, Supporting Information). TLR2 is a key sensor of mycobacterial lipoproteins; downstream MyD88 signaling is required for early control of mycobacterial infection and tunes NF‐κB‐dependent transcription in infected phagocytes. Thus, predicted binding near these interfaces suggests that punicalagin may allosterically modulate TLR2‐MyD88‐NF‐κB signaling, a pathway tightly coupled to antimicrobial effector deployment and autophagy crosstalk. MIF is a cytokine produced by macrophages and is known to play a role in both *M. tuberculosis* pathogenesis and host defense. Our docking results show that punicalagin binds near MIF's functional site, suggesting it may help dampen excessive inflammation without compromising antimicrobial responses. Furthermore, we found that punicalagin suppressed AKT/mTOR and MAPK signaling to promote autophagy‐lysosomal clearance of MAB while preserving mitochondrial architecture (Figure 3i). Additionally, punicalagin's efficacy may also involve activation of the SIRT1‐FoxO axis, suggesting a multi‐pathway, host‐directed therapeutic strategy (Figure 3m,n).

### 2.4 Punicalagin Treatment Upregulates SIRT1 and FoxO3a in MAB‐Infected Macrophages to Promote Autophagy

**Figure 4:**
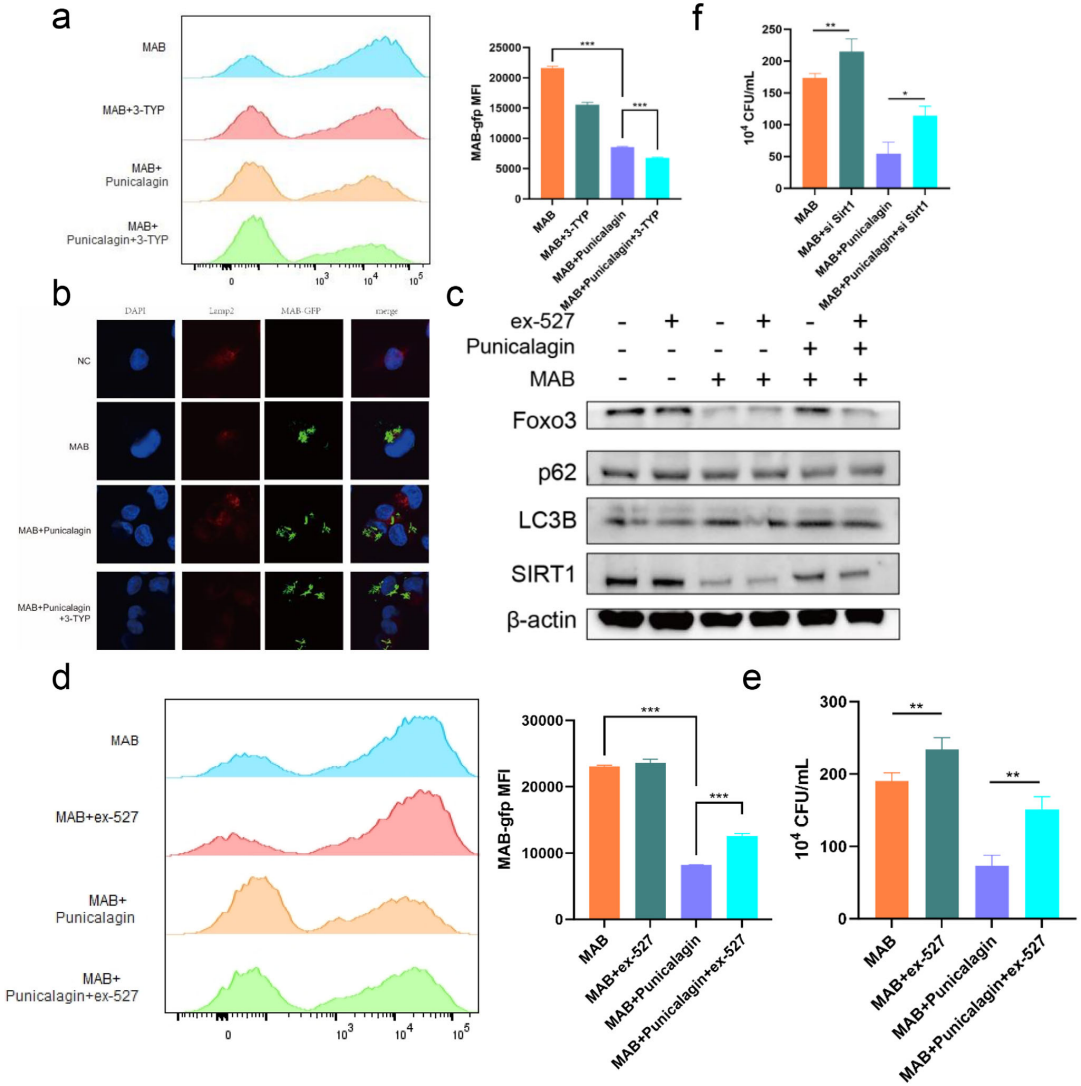
Punicalagin‐treatment upregulated autophagy‐related genes through SIRT1/FoxO3a pathway in MAB‐infected THP‐1 cells. a) Fluorescence intensity of intracellular MAB‐GFP with or without 3‐TYP. b) Co‐localization of MAB‐GFP with LC3B with or without 3‐TYP. c) Representative WB images of autophagy markers and SIRT1 in MAB‐infected THP‐1 cells with punicalagin with or without ex‐527. d) Fluorescence intensity of intracellular MAB‐GFP with or without ex‐527. e) Intracellular MAB treated with punicalagin with or without SIRT1 siRNA followed by CFU count. f) Intracellular MAB treated with punicalagin with or without SIRT1 siRNA followed by CFU count. Statistical significance is indicated as *p < 0.05; **p < 0.01; ***p < 0.001; ****p < 0.0001; ns denotes a non‐significant difference. n = 3.

SIRT1 and SIRT3, essential members of the Sirtuin family, play pivotal roles in regulating diverse biological processes through their deacetylation activity, impacting metabolic regulation, cell survival, stress responses, inflammation, and aging. To further examine whether punicalagin treatment regulates intracellular mycobacterial survival specifically via SIRT1 expression, we conducted experiments to eliminate the potential influence of SIRT3 using SIRT3 inhibitor 3‐TYP and SIRT1 inhibitor ex‐527. Flow cytometry analysis revealed that SIRT3 blockade did not attenuate punicalagin's antibacterial efficacy, as bacterial burden reduction remained comparable to punicalagin‐treated controls (**Figure** **4**a). Immunofluorescence results also showed no significant changes in autophagy in cells treated with 3‐TYP in response to MAB infection and punicalagin treatment (Figure 4b). However, treatment with SIRT1 inhibitor ex‐527 resulted in a reduction of autophagy‐related protein expression in punicalagin‐treated MAB‐infected THP‐1 cells when compared to untreated MAB‐infected cells. Additionally, there was a notable decrease in the expression levels of SIRT1 and FoxO3a (Figure 4c). Flow cytometry analysis demonstrated a decrease in phagocytic activity against MAB in punicalagin‐treated THP‐1 cells that were treated with SIRT1 inhibitor ex‐527. Quantitative assessment of intracellular MAB confirmed that ex‐527 compromised the capacity of SIRT1‐treated macrophages to effectively combat infection (Figure 4d,e). Our findings indicate that the SIRT1 inhibitor ex‐527 diminished the capacity of punicalagin to reduce the intracellular load of MAB within macrophages, rather SIRT3 inhibitor. These findings functionally ruled out SIRT3 as being involved in punicalagin's mechanism of action, reinforcing SIRT1 as the primary target orchestrating autophagy‐mediated pathogen clearance.

**Figure 5:**
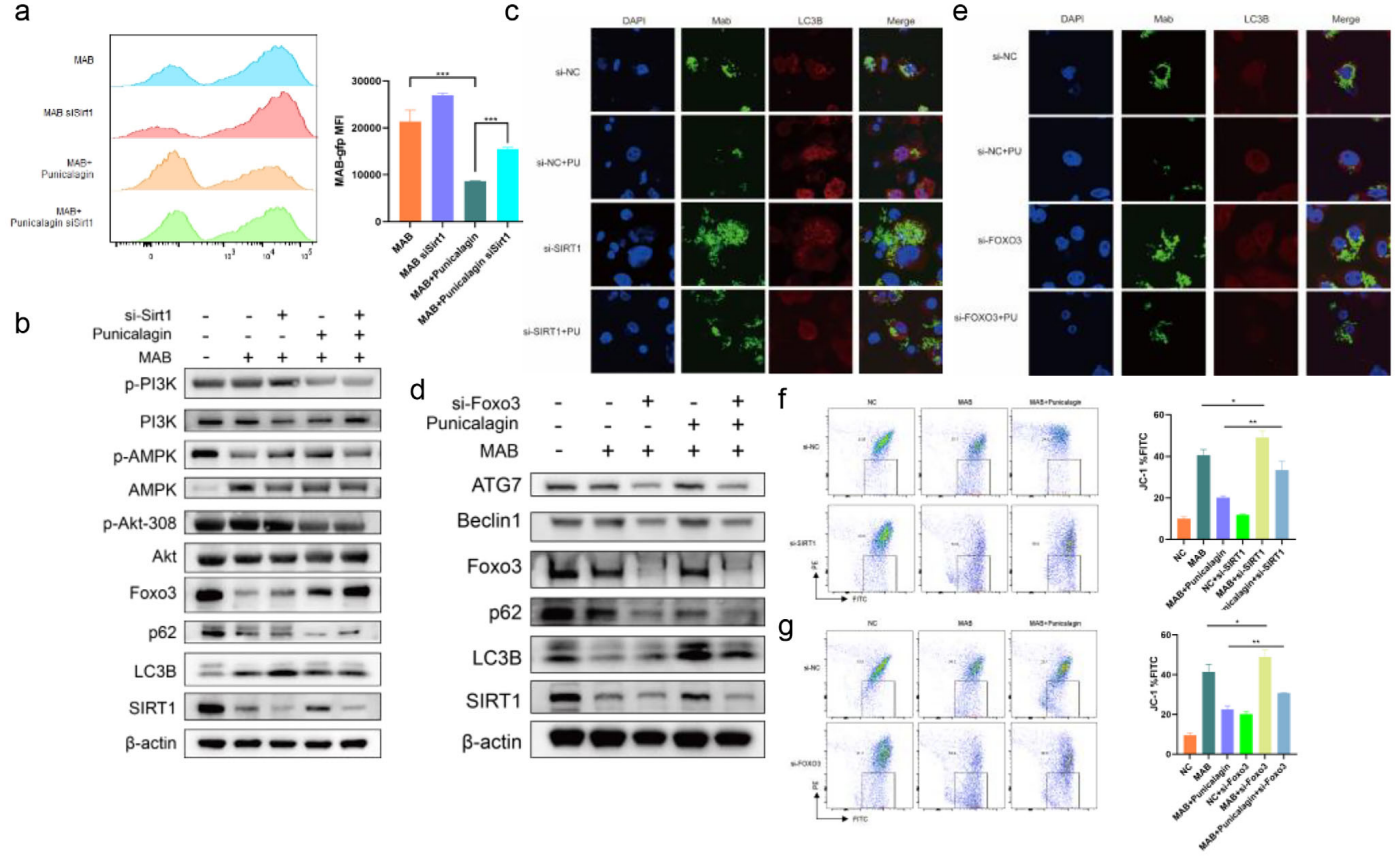
Punicalagin requires SIRT1/FoxO3a signaling to enhance host defense against MAB infection by restoring autophagic flux and mitochondrial homeostasis, whereas SIRT1/FoxO3a knockdown reverses this effect. (a) Fluorescence intensity of intracellular MAB‐GFP with or without si‐SIRT1. b) WB results of si‐SIRT1 knockdown or control treatment following infection. c) Localization of MAB‐GFP with or without si‐SIRT1 and punicalagin treatment. d) WB results of si‐FoxO3a knockdown or control treatment following infection. e) Localization of MAB‐GFP with or without si‐FoxO3a and punicalagin treatment. f) Flow cytometry analysis of JC‐1 staining to evaluate Δψm in cells after si‐SIRT1 knockdown or control treatment following infection. g) Flow cytometry analysis of JC‐1 staining to evaluate Δψm in cells after si‐FoxO3a knockdown or control treatment following infection. Statistical significance is indicated as *p < 0.05; **p < 0.01; ***p < 0.001; ****p < 0.0001; ns denotes a non‐significant difference. n = 3.

To further validate the critical function of SIRT1 in the clearance of MAB mediated by punicalagin, we conducted siRNA knockdown of SIRT1 in macrophages prior to the administration of punicalagin. The knockdown of SIRT1 in macrophages significantly diminished the ability of punicalagin‐treated THP‐1 cells to clear intracellular MAB (Figure 4f and **Figure** **5**a). Given the limitation of verifying knockdown efficiency by qRT‐PCR, we assessed SIRT1 expression by Western blot analysis, which indeed confirmed a reduction in SIRT1 protein levels following siRNA treatment. Additionally, we observed that the downregulation of SIRT1 expression altered punicalagin‐induced autophagy‐related functions in macrophages (Figure 5b). We investigated the impact of siRNA‐mediated SIRT1 knockdown on the colocalization of LC3B in THP‐1 cells infected with MAB‐GFP.

Our findings revealed that, MAB infection significantly impaired autophagy function in macrophages, leading to cell death and exacerbation of infection (Figure 5a–c, first row). However, punicalagin treatment effectively containment of GFP‐labeled MAB within autophagosome of infected THP‐1 (Figure 5c, second row). Notably, SIRT1 knockdown via si‐SIRT1 further aggravated MAB infection, as evidenced by the inability of THP‐1 cells to restrict intracellular MAB proliferation shown by increased amount of GFP‐labeled MAB (Figure 5a), significantly impaired autophagy function in macrophages, ultimately leading to cell death and exacerbation of infection as evidenced by increased nuclear fragmentation (Figure 5c, third row). Furthermore, punicalagin treatment attenuated the effect of enhanced MAB proliferation mediated by si‐SIRT1 knockdown within THP‐1 (Figure 5c, fourth row), demonstrating that SIRT1‐mediated pathways play a critical role in punicalagin‐enhanced autophagy mechanisms. These findings demonstrate that punicalagin's protective effect against MAB‐induced cellular damage is dependent on SIRT1 activation, which subsequently drives autophagy to facilitate MAB clearance. This underscores SIRT1 as a key therapeutic target for mitigating MAB infection and highlights punicalagin's potential as a SIRT1‐activating agent to enhance cellular defense mechanisms against intracellular MAB infection.

Building on evidence of FoxO3a upregulation and pathway enrichment in punicalagin‐treated macrophages, we next investigated the role of FoxO3a, a master transcriptional regulator of autophagy and stress adaptation in MAB‐infected macrophages. FoxO3a expression was silenced via siRNA in MAB‐infected THP‐1 macrophages prior to punicalagin treatment. We found that FoxO3a knockdown abrogated punicalagin‐induced autophagic flux, reduced ATG7 expression, a critical autophagy effector transcriptionally regulated by FoxO3a (Figure 5d). Imaging analysis of GFP‐labeled MAB co‐localized with LC3B demonstrated that punicalagin significantly suppressed intracellular MAB infection in THP‐1 cells (Figure 5e, second row). However, si‐FoxO3a treatment exacerbated the dispersion of GFP‐labeled MAB within THP‐1 cells (Figure 5e, third row) and abrogated the anti‐infective efficacy of punicalagin (Figure 5e, fourth row). Furthermore, FoxO3 knockdown impaired autophagosome formation (Figure 5d,e). Lastly, we evaluated the generation of mitochondrial membrane potential in cells treated with si‐SIRT1 and si‐FoxO3a using flow cytometry. Our results demonstrated that damage to the mitochondrial membrane potential was exacerbated in MAB‐infected THP‐1 cells subjected to si‐SIRT1 and si‐FoxO3a treatment (Figure 5f and g). Additionally, the protective effect of punicalagin on mitochondrial membrane potential in infected cells was significantly diminished in si‐SIRT1 or si‐FoxO3a groups (Figure 5f,g). These results indicate that punicalagin positively affects autophagic flux to inhibit intracellular MAB growth through upregulation of SIRT1 or FoxO3a. These findings mechanistically position the SIRT1‐FoxO3a axis as an essential signaling nexus through which punicalagin coordinates autophagy to enable autophagic pathogen clearance.

### 2.5 Punicalagin Treatment Enhances Host Defense Against MAB Infection in Mice

**Figure 6:**
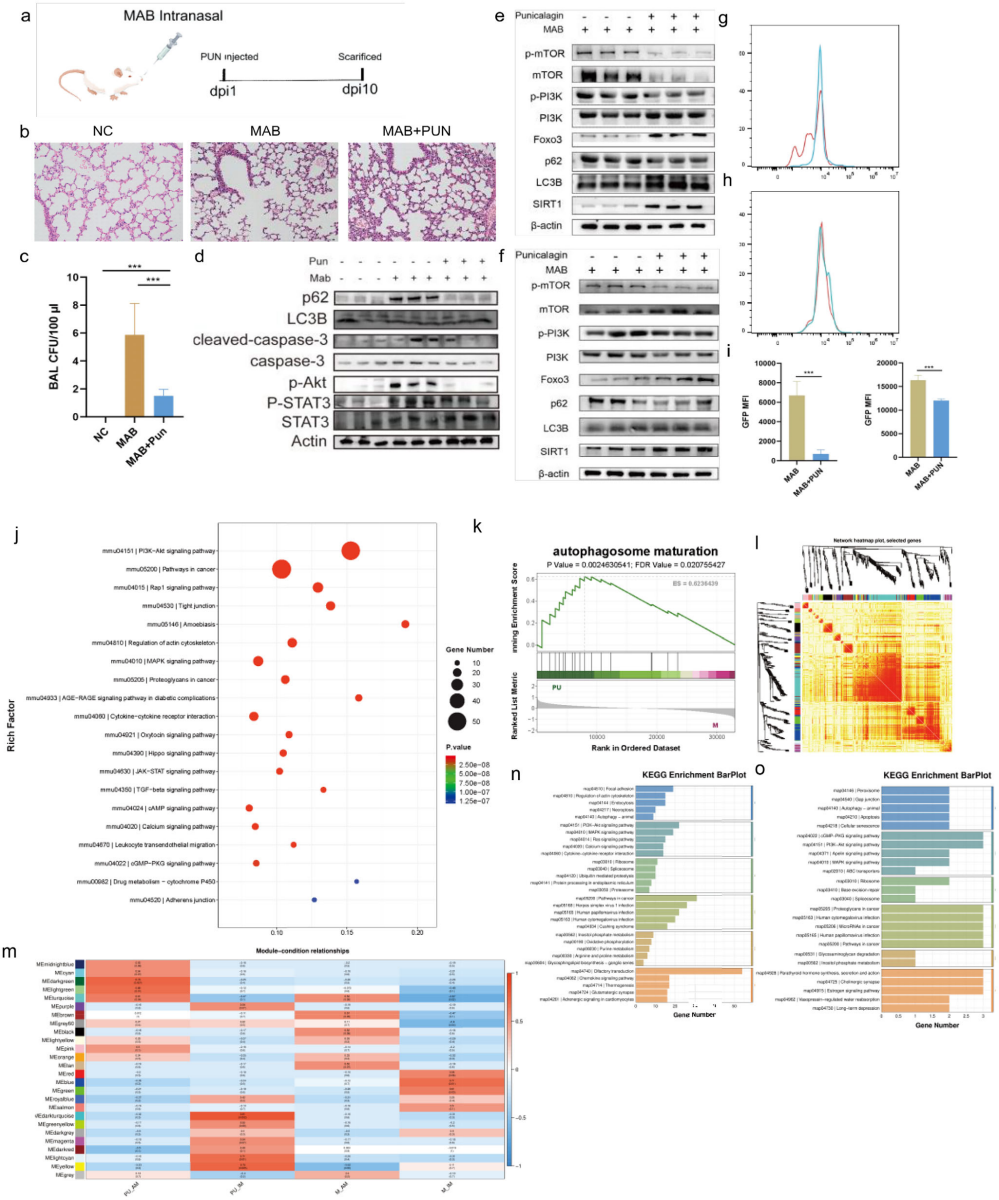
Treatment of MAB‐infected mice with punicalagin and pulmonary gene expression changes in response to MAB infection and punicalagin (PUN) treatment. a) Study design of the mouse experiment. b) Typical pictures of HE stains. c) CFU data expressed as CFU/100 ml for bacterial burdens in the lung. d) Lung WB results of autophagy‐related proteins. e) Interstitial macrophage WB results of autophagy‐related proteins. f) Alveolar macrophage WB results of autophagy‐related proteins. g) Representative image showing the mean fluorescence intensity of GFP‐MAB in interstitial macrophages (CD64+CD11b+) of mouse lungs. h) Representative image showing the mean fluorescence intensity of GFP‐MAB in alveolar macrophages (CD45+SiglecF+) of mouse lungs. i) fluorescence intensity of GFP‐MAB in interstitial macrophages (left) and alveolar macrophages (right). j) KEGG pathway enrichment analysis. k) GSEA enrichment analysis. l) WGCNA analysis of DEGs. m) The module‐trait relationships. n) Yellow modules were the most closely related modules to punicalagin‐treated interstitial macrophages. o) Light‐green modules were the most closely related modules to punicalagin‐treated interstitial macrophages and alveolar macrophages. Statistical significance is indicated as *p < 0.05; **p < 0.01; ***p < 0.001; ****p < 0.0001; ns denotes a non‐significant difference. n = 5.

Having established the SIRT1‐FoxO3a axis as the key mechanism underlying punicalagin‐induced autophagy‐dependent anti‐MAB activity in macrophages, we next evaluated its therapeutic efficacy in a murine model of pulmonary MAB infection. Mice were inoculated with 10^7^ CFU of MAB via intranasal instillation (**Figure** **6**a), followed by intraperitoneal administration of punicalagin. Comparative analysis between punicalagin‐treated MAB‐infected mice and untreated MAB‐infected mice revealed that during the infection period, the punicalagin treatment group exhibited milder inflammatory cell infiltration and reduced alveolar damage in the lung tissues (Figure 6b), accompanied by a significant reduction in lung BALF MAB CFU counts (Figure 6c).

We then assessed autophagy modulation and apoptosis regulation in the lung tissues of MAB‐infected mice and MAB‐infected mice treated with punicalagin. Punicalagin significantly inhibited STAT3 activation, notably suppressed AKT levels, and reduced cleaved caspase‐3 levels, indicating attenuated apoptosis alongside increased autophagic activity, a phenotype consistent with its cytoprotective effects observed ex vivo (Figure 6d).

To further investigate the effect of punicalagin on autophagy and related cell signaling pathways in different macrophage subsets, we isolated alveolar macrophages (SiglecF⁺) and interstitial macrophages (CD11b⁺) from the lungs of MAB‐infected mice, both untreated and punicalagin‐treated, using cell sorting techniques. Western blot analysis of autophagy‐related proteins, along with SIRT1 and FoxO3a, revealed that in the interstitial macrophages of punicalagin‐treated MAB‐infected mouse lungs, SIRT1 and FoxO3a expression were upregulated, accompanied by reduced PI3K/mTOR levels, effectively counteracting autophagy inhibition (Figure 6e). Interestingly, similar trends were observed in alveolar macrophages (Figure 6f), further supporting the broad applicability of punicalagin's host‐directed mechanism in modulating autophagy and combating MAB infection. To assess the therapeutic effects of punicalagin on various macrophage populations during MAB infection in the mouse lungs, we employed flow cytometry to evaluate the infection status of GFP‐MAB within lung macrophages. Alveolar macrophages were identified by the CD45⁺SiglecF⁺, while interstitial macrophages were characterized by the expression of CD45⁺CD64⁺CD11b⁺. Punicalagin treatment exhibited a stronger protective effect against MAB infection in interstitial macrophages than in alveolar macrophages, as evidenced by the marked reduction in bacterial burden (Figure 6g,i). While punicalagin also mitigated the bacterial burden in alveolar macrophages, the severity of infection remained notably higher in alveolar macrophages than interstitial macrophages (Figure 6h,i). This observation highlights the varying susceptibility of different macrophage populations to MAB infection and underscores punicalagin's selective effectiveness in targeting lung macrophages.

**Figure 7:**
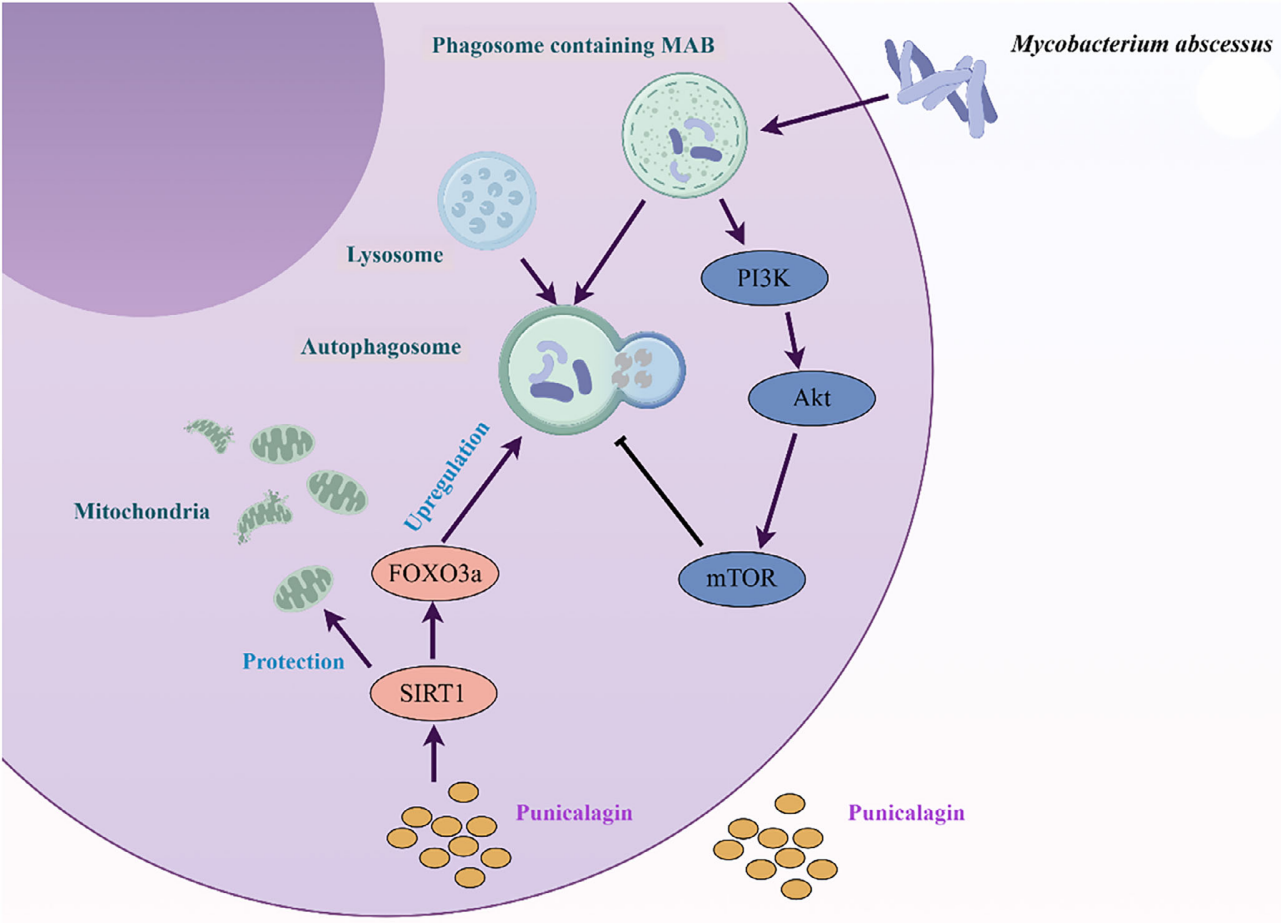
Schematic diagram depicts the mechanism of punicalagin‐mediated SIRT1 upregulation in counteracting MAB infection within macrophages. Punicalagin activates SIRT1, which upregulates FOXO3a, enhancing its transcriptional activity. The SIRT1/FOXO3a axis suppresses apoptosis and protects mitochondria from oxidative damage. Simultaneously, SIRT1 promotes autophagy, which is inhibited by the activation of the PI3K/Akt/mTOR pathway, enhancing autophagosome formation and phagosome‐lysosome fusion, reducing suppressed MAB‐containing phagosomes. Arrows denote activation or progression, illustrating punicalagin's role in boosting mitochondrial protection and autophagic clearance of intracellular MAB.

To investigate the differential effects of punicalagin on alveolar macrophages and interstitial macrophages infected with MAB, we conducted transcriptomic sequencing on flow‐sorted alveolar macrophages and interstitial macrophages from both MAB‐infected and punicalagin‐treated MAB‐infected groups. Notably, in punicalagin‐treated MAB‐infected lung macrophages, several keys signaling pathways and biological processes were significantly enriched in macrophages compared to MAB‐infected controls. These included the PI3K‐Akt signaling pathway, MAPK signaling pathway, calcium signaling pathway, FoxO signaling pathway, HIF‐1 signaling pathway, phagosome formation, Fc gamma receptor‐mediated phagocytosis, and endocytosis. These findings highlight the broad regulatory effects of punicalagin on cellular processes critical for immune response and pathogen clearance (Figure 6j). Given the more pronounced effects of punicalagin treatment in interstitial macrophages (Figure 6h,j), we performed Gene Set Enrichment Analysis (GSEA) comparing macrophages in the punicalagin‐treated infection groups. We found that the autophagosome maturation was significantly enriched in punicalagin‐treated interstitial macrophages (Figure 6k), indicating enhanced autophagic activity. This finding is consistent with our previous observations of SIRT1‐mediated activation of FoxO3a in THP‐1 macrophages (Figures 4 and 5), which promoted autophagy to combat the intracellular pathogen. The upregulation of autophagosome maturation suggests that punicalagin enhanced macrophage effector functions by facilitating the clearance of MAB through autophagy, thereby contributing to its therapeutic effects. To further investigate, we used Weighted Gene Co‐Expression Network Analysis (WGCNA) to construct a co‐expression network and identify modules associated with punicalagin treatment by clustering genes based on expression patterns. Our analysis identified two key modules (light‐green module and yellow module) strongly associated with the treatment group clusters in both interstitial macrophages and alveolar macrophages (Figure 6l,m). Specifically, significant enrichment of endocytosis, proteolysis, and autophagy processes (yellow module) were found in the interstitial macrophage treatment group, while only autophagy‐related processes (light‐green module) were enriched in the alveolar macrophages’ treatment group. KEGG enrichment analysis revealed that autophagy and lysosome‐related functions were significantly more enriched in interstitial macrophages than in alveolar macrophages in the punicalagin‐treated MAB‐infected mice (Figure 6n,o). The result highlights the differential effects of punicalagin on alveolar and interstitial macrophages in mice infected with MAB. The findings from this study demonstrate that punicalagin combats pulmonary MAB infection by activating the SIRT1‐FoxO3a axis. This activation suppresses PI3K/mTOR and STAT3 signaling pathways, thereby enhancing autophagy while reducing apoptosis. This host‐directed mechanism results in decreased lung inflammation and bacterial burden, with interstitial macrophages showing preferential response through autophagosome‐lysosome interactions. Transcriptomic profiling further underscores the macrophage subset‐specific engagement of autophagy‐lysosomal pathways, which aligns with punicalagin's therapeutic selectivity. By modulating macrophage‐intrinsic defenses rather than directly targeting MAB, punicalagin emerges as a promising candidate for the treatment of persistent mycobacterial infections (**Figure** **7**).

## 3 Discussion

NTM infections represent a significant public health challenge globally, including in developed nations, where their incidence often surpasses that of *M. tuberculosis*.^[^ ^8^, ^47^ ^]^ The treatment of NTM infections, particularly those caused by MAB remains suboptimal, underscoring the urgent need for novel antimicrobials and therapeutic agents to combat this pathogen. Host‐directed therapy (HDT) aims to enhance host immunity or target interactions in the pathogen‐host interplay, providing a framework for identifying drugs that can counteract mycobacterial immune evasion mechanisms. However, limited knowledge exists regarding potential candidate drugs capable of activating host immune responses and cellular phagocytosis against NTM. In this study, we demonstrate that punicalagin is a promising HDT agent for treating MAB infection, exerting its effects without direct bactericidal activity. Our findings indicate that punicalagin mediates its anti‐MAB effects through a series of interconnected mechanisms. Specifically, it upregulates the expression of SIRT1 and FoxO3a, mitigating mitochondrial damage while enhancing autophagy and phagosome maturation. Our study identifies punicalagin as a HDT candidate that suppresses intracellular MAB survival through SIRT1/FoxO3a‐dependent mitochondrial protection and autophagic reprogramming, with preferential efficacy in interstitial macrophages.

Punicalagin, a major polyphenolic compound found in pomegranates, is present in the fruit, leaves, and peel of the plant.^[^ ^48^, ^49^ ^]^ Additionally, there are reports that punicalagin promotes autophagy by activating the MAPK pathway and inhibiting the mTOR signaling pathway, thereby inducing apoptosis in tumor cells, and exhibiting potential anti‐tumor effects.^[^ ^50^, ^51^ ^]^ Some studies also indicate that high concentrations of punicalagin (10 mg/ml) exhibit antibacterial activity in vitro, inhibiting the growth of pathogens such as *E. coli*, However, the in vivo relevance and safety of such concentrations remain unclear.^[^ ^52^ ^]^ However, whether punicalagin can inhibit mycobacteria both in vitro and in vivo is unknown. Our research identifies punicalagin as a promising therapeutic agent against MAB infections through host‐directed mechanisms. In contrast to previous studies that primarily focused on its anti‐tumor and general antibacterial properties, our findings indicate that punicalagin enhances intracellular defense mechanisms against MAB without direct bactericidal activity. Specifically, it upregulates the SIRT1/FoxO3a axis, reducing oxidative stress and mitochondrial damage while simultaneously promoting autophagy and phagosome maturation.

Autophagy serves as a critical host‐bacteria defense mechanism and is a target for HDT in combating mycobacterial infections.^[^ ^13^, ^53^ ^]^ Several autophagy‐enhancing agents demonstrate activity against intracellular MAB in macrophages in preclinical models. V46 and amiodarone promote nuclear translocation of transcription factor EB (TFEB), V46 inhibiting Akt‐mTOR signaling and activating AMPK, thereby enhancing autophagosome‐lysosome fusion, and facilitating intracellular MAB clearance.^[^ ^33^, ^54^ ^]^ Gemfibrozil activates PPARα, which upregulates TFEB‐dependent autophagy and reduces inflammatory pathology, contributing to improved control of MAB within macrophages.^[^ ^55^ ^]^ N‐degron mimetics engage the SQSTM1/p62 to recruit LC3 and target intracellular MAB for selective autophagic degradation in an mTOR‐independent manner.^[^ ^56^ ^]^ Phosphatidylserine liposomes and ohmyungsamycin A also enhance macrophage‐mediated killing of MAB through phagosome acidification and reactive oxygen species production, or via mitochondrial ROS‐driven M1 polarization.^[^ ^57^ ^]^ In this context, punicalagin contributes to MAB control by supporting autophagic clearance through coordinated effects on host defense mechanisms, warranting further study as a potential autophagy‐targeting agent. During infections with MAB, the antimicrobial action of punicalagin is primarily mediated through multiple host defense strategies. Punicalagin helps maintain intracellular homeostasis by reducing mitochondrial damage in the body and within macrophages during infection. Previous research indicates that SIRT1 plays a key role in regulating cellular oxidative stress and its associated toxicity.^[^ ^58^, ^59^ ^]^ As a SIRT1 activator, punicalagin enhances SIRT1's dual capacity to orchestrate mitochondrial safeguarding and autophagy initiation through the SIRT1/FoxO3a axis, positioning SIRT1‐mediated mitochondrial protection and autophagic flux as central pillars of its host‐directed therapeutic strategy (Figures 3i and 4c). Mechanistically, SIRT1 deacetylates FoxO3a transcription factor, enhancing its transcriptional activity to upregulate antioxidant enzymes that counteract oxidative stress and suppress apoptosis.^[^ ^58^ ^]^ Intriguingly, the SIRT1‐ FoxO3a interaction exhibits bidirectional regulation: while SIRT1 deacetylates FoxO3a to amplify its cytoprotective functions, FoxO3a reciprocally enhances SIRT1 transcription by binding to the SIRT1 promoter in a p53‐dependent manner, forming a self‐reinforcing antioxidant loop.^[^ ^60^ ^]^ FoxO3a reciprocally amplifies autophagy by transcriptionally activating core autophagy genes (e.g., LC3, ATG7), creating a synergistic mechanism for pathogen clearance (Wu et al., 2024). This interplay explains how punicalagin, as a SIRT1 agonist, enhances phagosome‐lysosome fusion in MAB‐infected macrophages and facilitates both autophagosome maturation and lysosomal function (Figure 3c). The PI3K/Akt/mTOR signaling pathway, a key pathway enhanced during intracellular mycobacterial infection, plays a significant role in modulating autophagy.^[^ ^47^ ^]^ By activating the SIRT1/FoxO3a axis, punicalagin could override pathogen‐driven mTOR signaling, redirecting host machinery toward antimicrobial autophagy while concurrently shielding mitochondria from infection‐associated oxidative damage. Our study further explored how punicalagin might broadly modulate host immunity. Integrated proteomic and in silico molecular docking analyses revealed favorable binding between punicalagin and several key innate immune proteins upregulated during infection, including Toll‐like receptor 2 (TLR2), its adaptor MyD88, and the cytokine macrophage migration inhibitory factor (MIF). The predicted high‐affinity interaction at the TLR2‐MyD88 interface suggests that punicalagin may modulate NF‐κB signaling, a pathway linked to antimicrobial effector functions and autophagy. Although experimental validation is needed, these findings indicate that punicalagin's therapeutic effect likely extends beyond SIRT1 activation to include direct engagement with multiple components of the innate immune system.

Studies indicate that MAPK/ERK signaling enhances STAT3 transcription, while SIRT1 suppresses STAT3 activity via deacetylation and reduced phosphorylation.^[^ ^48^ ^]^ In our study, punicalagin inhibited MAPK pathway activation and concurrently suppressed STAT3 activity in MAB‐infected mouse lung tissue, through dual mechanisms: promoting SIRT1 upregulation to mitigate MAPK‐driven oxidative stress and directly impair STAT3 activation. In mycobacterial infections, STAT3 activation promotes intracellular pathogen survival by reinforcing anti‐apoptotic responses and suppressing autophagy via IL‐10‐mediated pathways and transcriptional regulation of autophagy‐related genes.^[^ ^49^ ^]^ Punicalagin's disruption of STAT3 activation liberates the autophagy machinery from transcriptional repression while synergizing with SIRT1‐dependent deacetylation in mitochondria to restore mitochondrial stability and counteract MAB infection (Figure 6d).

Previous studies on *M. tuberculosis* have revealed that the response of infected alveolar macrophages involves a metabolic shift from oxidative phosphorylation to HIF‐1α‐driven Warburg glycolysis, coupled with NOS2 induction and NF‐κB‐mediated proinflammatory activation, a program designed to enhance bactericidal capacity.^[^ ^61^ ^]^ In alveolar macrophages, this Warburg‐like glycolytic shift exhibits a paradoxical duality, while boosting antimicrobial activity via reactive nitrogen species and inflammatory cytokine production, it concurrently elevates fatty acid oxidation, creating a lipid‐rich microenvironment that inadvertently fuels mycobacterial replication.^[^ ^62^, ^63^ ^]^ However, in this study, we found that interstitial macrophages exhibited superior antimicrobial capacity compared to alveolar macrophages during MAB infection (Figure 6n). Interstitial macrophages restrict pathogen growth through pro‐inflammatory and autophagy pathways.^[^ ^16^ ^]^ Restoring interstitial macrophage's function, particularly enhancing autophagy, could counter MAB proliferation. Our findings reveal that interstitial macrophages are the preferentially more important cells affected by punicalagin via SIRT1‐FoxO3a‐mediated autophagy and phagosome maturation, thereby effectively counteracting MAB's immune evasion tactics. The preferential targeting of interstitial macrophages is therapeutically advantageous, as these cells limit MAB infection. Transcriptomic profiling revealed that punicalagin‐treated interstitial macrophages displayed pronounced enrichment of autophagy‐lysosomal pathways (Figure 6j,k), including autophagosome maturation and lysosomal biogenesis, alongside metabolic rewiring toward oxidative phosphorylation, a process regulated by SIRT1‐FoxO3a‐mediated mitochondrial stabilization, while alveolar macrophages activated only the autophagy‐related module. By enhancing interstitial macrophages’ capacity to degrade pathogens while restraining STAT3/PI3K‐driven inflammation, punicalagin resists infection without exerting direct bactericidal effect. This macrophage‐subset‐specific reprogramming underscores the potential of host‐directed therapy to outmaneuver intracellular pathogens by leveraging inherent immune cell plasticity rather than targeting pathogen‐specific vulnerabilities. By activating the SIRT1, punicalagin restores dysregulated autophagic flux during MAB infection, which is tightly linked to mitochondrial membrane stabilization and enhances intracellular pathogen clearance (Figure 4d). This pathway modulation not only counteracts pathogen‐driven immune evasion but also reverses the suppression of autophagy in macrophages, as evidenced by improved mitochondrial integrity and upregulated autophagic activity. Importantly, our findings align with recent reports highlighting SIRT1/FoxO3a as a central hub for resolving immune‐metabolic dysfunction in arthritis or hypoxia‐ischemia brain injury,^[^ ^64^, ^65^ ^]^ yet uniquely extend this paradigm to infectious contexts by demonstrating punicalagin's dual capacity to reinforce mitochondrial resilience and reinvigorate host autophagy to facilitate pathogen clearance. Further clinical studies are needed to assess the role of punicalagin as an HDT agent in facilitating the clearance of MAB infection in patients. Although this study attributes the primary anti‐MAB effects to punicalagin, its partial conversion to ellagic acid was observed. We recognize that ellagic acid may also modulate host macrophage responses. Therefore, future studies are warranted to explore the distinct effects of ellagic acid on autophagy, which would help clarify any potential roles and fully delineate the therapeutic activities of pomegranate‐derived metabolites in controlling MAB infection.

## 4 Conclusion

This study highlights the therapeutic potential of punicalagin in modulating host responses during MAB infection. Our findings indicate that punicalagin significantly enhances the autophagic function of macrophages, thereby reducing the intracellular survival of MAB. Mechanistically, punicalagin exerts its effects by upregulating SIRT1 and FoxO3a. Furthermore, punicalagin inhibits the PI3K/Akt/mTOR signaling pathway, promoting autophagic flux and facilitating bacterial clearance. In vivo mouse experiments demonstrated that punicalagin reduces inflammation and bacterial burden in the lungs of MAB‐infected mice. Despite these promising findings, additional research is needed to investigate the long‐term efficacy and safety of punicalagin in clinical settings. By modulating macrophage‐intrinsic defenses rather than directly targeting MAB, punicalagin may serve as a promising candidate for the treatment of persistent mycobacterial infections.

## 5 Experimental Section

### Cell Culture

The THP‐1 cell line was bought from the Shanghai Cell Bank of the Chinese Academy of Sciences in 12/2021 (Shanghai, China, SCSP‐567, RRID: CVCL_0006) and cultured in RPMI 1640 medium (Gibco, C11875500BT,) supplemented with 1% penicillin‐streptomycin (Gibco, 15 140 122). All cells were maintained at 37 °C in a 5% CO_2_ atmosphere. For mycobacterial infection assays, antibiotics were excluded from the culture medium to avoid interference with pathogen viability. Differentiation into macrophages was induced by treating THP‐1 monocytes with 20 ng mL^−1^ phorbol 12‐myristate 13‐acetate (PMA) (Sigma‐Aldrich) for 16 h. In the main text, PMA‐differentiated THP‐1 cells are referred to as THP‐1 macrophages.^[^ ^66^ ^]^ Bone marrow‐derived macrophages (BMDM) were isolated from the femur of Balb/c mice as described. After exercising the bones, surface skin and muscles were removed, and the bone marrow was flushed using RPMI 1640 containing 10% fetal bovine serum (FBS).^[^ ^67^ ^]^ Following lysis of red blood cells and filtration through a 70‐µm mesh, the isolated cells were cultured in RPMI 1640 supplemented with 10% FBS, 1% penicillin‐streptomycin, and 40 ng/mL macrophage colony‐stimulating factor (M‐CSF) (PeproTech, 315‐02). The culture medium was refreshed on the third day, and cells were utilized for experiments on the seventh day.

### Bacterial Culture

*Mycobacterium abscessus* strain ATCC 19 977 (type strain) was obtained from the American Type Culture Collection (ATCC) and was cultured in Middlebrook 7H9 broth (BD Difco) supplemented with 0.05% Tween‐80, 0.2% glycerol, and 10% OADC enrichment at 37 °C with shaking for 3 days to stationary phase as previously described. The cultures were harvested, resuspended in phosphate‐buffered saline (PBS) containing 0.05% Tween 20 and 25% glycerol, and subsequently stored at −80 °C. Colony‐forming units (CFUs) per milliliter were quantified from a thawed stock vial.^[^ ^68^ ^]^

### Human Peripheral Blood Sample Collection

Peripheral blood samples were collected from 15 patients clinically diagnosed with *Mycobacterium abscessus* infection at the First Affiliated Hospital of Zhejiang University School of Medicine between January to December 2023. Following exclusion of 11 patients due to NGS‐confirmed co‐infections with other mycobacterial, bacterial, or fungal pathogens, or the presence of severe comorbidities (e.g., malignancies or HIV/AIDS), four previously untreated patients with confirmed MAB infection were included in the study. Age‐ and sex‐matched healthy individuals were recruited as controls. The study was approved by the clinical research ethics committee of the First Affiliated Hospital, College of Medicine, Zhejiang University (2023‐0100), and written informed consent was obtained from all participants.

### Non‐Targeted Metabolomics

Metabolite extraction was performed by combining 100 µL of THP‐1 cell culture supernatant with or without punicalagin with 400 µL of ice‐cold solvent mixture (acetonitrile: methanol, 1:1 v/v) containing 0.02 mg mL^−1^ L‐2‐chlorophenylalanine (internal standard) in 1.5 mL microcentrifuge tubes. Following homogenization via vortex mixing (30 s) and low‐temperature sonication (40 kHz, 5 °C, 30 min), protein precipitation was achieved by incubation at −20 °C for 30 min. The mixture was centrifuged (13 000×*g*, 15 min, 4 °C), after which the supernatant was collected, evaporated under nitrogen gas, and reconstituted in 100 µL of acetonitrile: water (1:1v/v). The reconstituted samples underwent additional sonication (40 kHz, 5 °C, 5 min) and centrifugation (13000×*g*, 10 min, 4 °C), with the final supernatant transferred to LC‐MS vials. Chromatographic separation was executed on Thermo UHPLC‐Q Exactive HF‐X system using an ACQUITY HSS T3 column with mobile phases of 0.1% aqueous formic acid (solvent A) and 0.1% formic acid in acetonitrile: isopropanol (1:1 v/v) at 0.4 mL/min (40 °C). Raw LC‐MS/MS data were processed through Progenesis QI to generate a 3D matrix in CSV format, followed by removal of internal standards, column artifacts, and noise‐derived peaks. Metabolite annotation was achieved by cross‐referencing the HMDB, Metlin, and Majorbio databases, with rigorous peak alignment and consolidation to eliminate redundancy.^[^ ^69^ ^]^

### Chemicals and Reagents Preparation

The chemicals Punicalagin (Aladdin, P117962, CAS 65995‐63‐3, ≥98% purity), 3‐TYP (MedChemExpress, HY‐108331, 40 µm, CAS 120241‐79‐4, 99.96% purity), and Chloroquine (MedChemExpress, HY‐17589A, 100 µm, CAS 54‐05‐7, 99.50% purity) were dissolved in dimethyl sulfoxide (Solarbio, D8371, CAS 67‐68‐5, ≥99.5% purity), while ex‐527 (MedChemExpress, HY‐15452, 10 µm, CAS 49843‐98‐3, 99.85% purity), Bafilomycin A1 (MedChemExpress, HY‐100558, 20 µM, CAS 88899‐55‐2, 99.95% purity), amikacin (MedChemExpress, BAY 41–6551, CAS 37517‐28‐5, 99.61% purity), Ellagic acid(MedChemExpress, HY‐B0183, CAS 476‐66‐4, 99.75% purity) were dissolved in double‐distilled water. All solutions were aliquoted and stored at −20 °C or −80 °C.

### Apoptosis Detection

Annexin V‐FITC/PI Apoptosis Kit (Liankebio, AT101) was used to assess apoptosis. THP‐1 macrophages were treated with 5 µL of FITC annexin V and 10 µL of PI, stained for 5 min. Apoptosis rates were analyzed using the FACSCanto II Flow Cytometry System (BD Biosciences, USA).^[^ ^70^ ^]^

### MAB Infection of THP‐1 Cells and Determination of Intracellular Survival

THP‐1 macrophage cells were seeded at a density of 5 × 10⁵ cells per well in a 12‐well plate and pretreated with MAB at a multiplicity of infection (MOI) of 10. The infected cells were incubated for 3 h, after which the medium was replaced, and the cells were co‐cultured with 50 µg mL^−1^ amikacin and punicalagin (40 µm) for an additional 24 h. Following this incubation, the cells were washed three times with pre‐warmed, sterile phosphate‐buffered saline (PBS) to eliminate extracellular bacteria. Subsequently, the cells were lysed using PBS containing 0.1% Triton X‐100, and the lysates were serially diluted and plated onto Middlebrook 7H10 agar (BD Biosciences, catalog number 262 710) supplemented with 10% oleic acid, albumin, dextrose, and catalase (OADC) and 0.5% glycerol. The plates were incubated at 37 °C for 3 days, after which bacterial colonies were counted, and the colony‐forming units (CFUs) were calculated.^[^ ^71^ ^]^

### Establishment of GFP‐Expressing *Mycobacterium abscessus*

The plasmid pTEC15 was obtained from Addgene (RRID: Addgene_30 174, https://www.addgene.org/30174/).^[^ ^72^ ^]^ *Mycobacterium abscessus* was prepared as previously described and inoculated into 5 mL of 7H9 liquid medium, followed by incubation at 37 °C with shaking until reaching the logarithmic phase. The bacterial cells were then pelleted by centrifugation at 4 °C (4000×*g*, 10 min) and resuspended at high concentration. The pTEC15‐GFP plasmid (harboring a hygromycin resistance gene) was adjusted to a concentration of ≥100 ng µL^−1^. A volume of 100 µL of the MAB suspension was mixed with an appropriate amount of plasmid DNA and incubated on ice for 5 min. The mixture was transferred to an electroporation cuvette and subjected to electroporation using the following parameters: 2.5 kV, 25 µF, 1000 Ω. Immediately afterward, 1 mL of 7H9 medium was added, and the culture was incubated at 37 °C without shaking for 2–3 h, then transferred to a shaker. After centrifugation, the bacterial suspension was plated onto 7H11 agar plates containing hygromycin and incubated at 37 °C until single colonies appeared.^[^ ^73^ ^]^

### Flow cytometry Analysis of MAB‐GFP‐Infected Cells Treated with Punicalagin

THP‐1 macrophages were seeded at 5 × 10^5^ cells per well in a 12‐well plate and pretreated with PMA for 24 h. The cells were infected with GFP‐labeled MAB‐GFP following the method described above, with or without punicalagin‐treatment (0, 20, 40, 80 µm) and washed three times with warm PBS to remove extracellular bacteria. The collected cells were transferred to fluorescence‐activated cell sorting (FACS) tubes, and the percentages of GFP‐positive cells were measured using the FACSCanto II Flow Cytometry System (RRID:SCR_01 8056, BD Biosciences, USA) after a 24‐h infection. Data were analyzed with FlowJo X 10.0.7 software according to the manufacturer's protocol.^[^ ^74^ ^]^

### Measurement of Mitochondrial Membrane Potential (ΔΨm)

THP‐1 macrophages were seeded at 2 × 10^5^ cells per well in a 12‐well plate and pre‐treated with punicalagin (40 µm) and MAB following the method described above. Extracellular bacteria were removed by washing three times with warm PBS. According to the manufacturer's instructions, the generation of mitochondrial membrane potential △Ψm of THP‐1 macrophages was quantified using JC‐1 dye. Fluorescence intensities of mitochondria within the cells were measured by FACSCanto II Flow Cytometry System (RRID:SCR_01 8056, BD Biosciences, USA) and analyzed by FlowJo X 10.10.0 according to the manufacturer's protocol.^[^ ^75^ ^]^

### RNA Interference And Transfection of THP‐1 Macrophages

THP‐1 cells were transfected with SIRT1 small interfering RNA (siRNA) (Genepharma, China) or negative control siRNA (GenePharma, China) using Invitrogen Lipofectamine 3000 (ThermoFisher, 13 778 075) according to the manufacturer's protocol. After 18 h, cells were differentiated by PMA and seeded at 5 × 10^5^ cells per well in a 12‐well plate. Following SIRT1 gene knockdown using siRNA, flow cytometry analysis, CFU counting, and Western blotting were performed to evaluate the effects of SIRT1 knockdown.^[^ ^75^ ^]^

### Western Blot

Animal tissues and THP‐1 cells were harvested and lysed in RIPA (Radioimmunoprecipitation assay) lysis buffer (Beyotime, China) for 20 min on ice. The protein concentration of the lysates was measured with a BCA protein assay kit (Beyotime, P0010S). Equal amounts of protein from each sample were separated by SDS‐PAGE and transferred onto PVDF membranes. The membrane was blocked with 5% skim milk powder in TBS (Tris‐buffered saline) with 0.1% Tween 20 for 1 h at room temperature and incubated with primary antibodies overnight at 4 °C. After washing, the membranes were incubated with appropriate secondary antibodies at room temperature for 1 h and visualized using ECL detection solution (Beyotime, P0018AS). The digital images of the protein bands were acquired using a ChemiScope 6000 (Clinx Science Instruments Co., Ltd, China).^[^ ^75^ ^]^ The primary antibodies used in the present study were anti‐LC3 (RRID:AB_1 079 382, Sigma, L8918), anti‐caspase‐3 (Apoptosis Antibody Sampler Kit, CST, 9915T), and anti‐β‐actin (RRID:AB_3 065 551, ABclonal, AC043), anti‐p62 (RRID:AB_2 862 742, ABclonal, A19700), anti‐STAT3 (RRID:AB_2 772 423, ABclonal, A16975), anti‐LAMP1 (RRID:AB_626 853, Santa Cruz, sc‐20011), anti‐Akt (RRID:AB_2 862 411, ABclonal, A18675), anti‐p‐Akt (RRID:AB_2 770 898, ABclonal, AP0637), anti‐mTOR (RRID:AB_2 764 355, ABclonal, A2445), anti‐p‐mTOR (RRID:AB_2 832 985, ABclonal, AP1413), Goat anti‐Rabbit IgG (H+L) Alexa Fluor 488 (RRID:AB_143 165, Thermo Fisher, A‐11008), Rabbit anti‐Mouse IgG (H+L) Alexa Fluor 555 (RRID:AB_2 535 848, Thermo Fisher, A‐21427), Goat anti‐Rabbit IgG (H+L) Alexa Fluor 555 (RRID:AB_141 784, Thermo Fisher, A‐21428), anti‐SIRT1 (RRID:AB_10 646 436, Proteintech, 13161‐1‐AP), anti‐SIRT3 (RRID:AB_2 239 240, Proteintech, 10099‐1‐AP), anti‐PI3K (RRID:AB_10 734 439, Proteintech, 20584‐1‐AP), anti‐p‐PI3K (RRID:AB_11 042 594, Proteintech, 60225‐1‐Ig), anti‐p‐STAT3 (RRID:AB_2 863 810, ABclonal, AP0705), anti‐Erk (RRID:AB_390 779, CST, 4695), anti‐p‐Erk (RRID:AB_2 315 112, CST, 4370), anti‐FoxO3a (RRID:AB_2 247 214, Proteintech, 10849‐1‐AP), anti‐ATG7 (RRID:AB_10 831 194, CST, 8558), anti‐AMPK (RRID:AB_330 331, CST, 2532), anti‐p‐AMPK (RRID:AB_331 250, CST, 2535)

### Quantitative Real‐Time PCR (qRT‐PCR)

Total RNA from THP‐1 cells and animal tissues was extracted using TRIzol reagent and then reverse‐transcribed into cDNA with HiScript III RT SuperMix for qPCR (Vazyme Biotech, China). RT‐qPCR analysis was performed with SYBR Green Master Mix (Vazyme Biotech, China).^[^ ^76^ ^]^ The primers recommended by the primer bank (https://pga.mgh.harvard.edu/cgi‐bin/primerbank/) were used and validated with NCBI primer blast (https://www.ncbi.nlm.nih.gov/tools/primer‐blast/). The primer sequences are listed in Table S1 (Supporting Information) of the supplementary materials.

### Mycobacterial Infection of Mice

Female BALB/c mice, aged 6–8 weeks, were obtained from Zhejiang Academy of Medical Sciences (RRID: MGI:2 683 685, Hangzhou, China). The animals were housed in specific‐pathogen‐free (SPF) facilities at the First Affiliated Hospital, Zhejiang University School of Medicine, maintained under controlled environmental conditions: temperature at 22 ± 1 °C, humidity between 50–60%, and a 12‐hour light/dark cycle. MAB (ATCC 19 977) was cultured in Middlebrook 7H9 broth supplemented with 10% OADC at 37 °C while shaking for 3 days to stationary phase. For infection, BALB/c mice (n = 5) were intranasally inoculated with 1 × 10⁷ colony‐forming units (CFUs) in 50 µL of saline under anesthesia.^[^ ^77^ ^]^ Infection progress was monitored after treatment initiation. Punicalagin, dissolved in phosphate‐buffered saline (PBS), was administered to mice via intraperitoneal injection at a daily dose of 20 mg kg^−1^ day^−1^ for 9 days, after which the animals were sacrificed. The study was approved by the animal care and use committee of the First Affiliated Hospital, College of Medicine, Zhejiang University (protocol number: 2023–938).

### Histopathology Study

Hematoxylin and eosin (H&E) staining was performed following standard protocols. Lung tissue samples were first fixed in 10% paraformaldehyde, embedded in paraffin, and then sectioned. Subsequently, the sections were stained with hematoxylin for five min followed by eosin staining for one minute. After staining, the tissue sections were dehydrated. Finally, all sections were mounted and examined using a brightfield microscope (RRID:SCR_01 8949, Olympus BX51, Tokyo, Japan) at a magnification of 200×.^[^ ^78^ ^]^

### Immunofluorescence Staining

Immunofluorescence staining was conducted at room temperature. Cells were fixed with 4% paraformaldehyde and permeabilized with 0.1% Triton X‐100 for 15 min. The cells were then blocked with 3% BSA for 30 min. Primary antibodies LC3B monoclonal antibody (RRID: AB_1 079 382, 1:100, Sigma), LAMP1 polyclonal antibody (RRID: AB_626 853, 1:100, Santa Cruz), SIRT1 rabbit monoclonal antibody (RRID: AB_626 853, 1:200, Proteintech) were diluted in blocking buffer and the incubation was conducted overnight at 4 °C. The cells were washed three times with PBS, each washing lasting 3 min. Cells were incubated with secondary antibodies goat anti‐rabbit IgG H&L Alexa Fluor 488 (RRID: AB_143 165, 1:500, Thermo Fisher), goat anti‐mouse IgG H&L Alexa Fluor 555 (RRID: AB_141 784, 1:500, Thermo Fisher) in the dark for 1 h. After secondary antibody incubation, cells were washed three times with PBS, each for 5 min. The nuclei were stained with DAPI for 5 min, followed by three additional PBS washes, each lasting 5 min.^[^ ^79^ ^]^ A fluorescent antifade agent was applied before observation under a laser confocal microscope (Olympus FV3000, Japan).

### Transmission Electron Microscopy

For TEM analysis of MAB‐infected cells, the samples were first washed with PBS and then fixed in 2.5% glutaraldehyde (v/v) prepared in 0.05 m sodium cacodylate‐HCl buffer (pH 7.2) at 4 °C for 2–4 h. Following fixation, standard processing procedures were performed. The sections were examined with a Hitachi transmission electron microscope (Japan) at an accelerating voltage of 80 kV.^[^ ^9^ ^]^

### Molecular Docking

Structures of punicalagin (α/β) and ellagic acid were retrieved from PubChem as SDF files and converted to PDB with Open Babel 2.3.2. Expected protonation/tautomeric states at pH 7.4 were inspected, and low‐energy conformers were generated and minimized (MMFF94). Ligands were prepared in AutoDockTools by adding polar hydrogens, assigning Gasteiger charges, defining rotatable bonds, and exporting as PDBQT. Target protein structures were downloaded from the RCSB PDB. Crystallographic waters and non‐essential ligands were removed in PyMOL 2.3.4 (essential metal ions/cofactors retained), after which receptors were protonated and charged in AutoDockTools and saved as PDBQT. Docking was performed with AutoDock Vina 1.1.2 using site‐focused grids centered on orthosteric or annotated pockets. Top‐ranked poses were evaluated for chemical plausibility; protein–ligand contacts (hydrogen bonds, salt bridges, π–π/π–cation, and hydrophobic interactions) were quantified with PLIP and visualized in PyMOL.^[^ ^80^ ^]^

### Statistical Analysis

GraphPad Prism 10.1.2 was used for data analysis. The results are presented as means ± standard deviations. A two‐tail paired or unpaired *t*‐test was utilized to evaluate statistically significant differences between the two groups. One‐way ANOVA was used to determine statistically significant differences among multiple groups. Tests were considered statistically significant when P values were <0.05. All experiments were conducted three times.

## Conflict of Interest

The authors declare no conflict of interest.

## Author Contributions

Y.Z. and K.X. designed and supervised the project; K.B. performed the experiments, analyzed the data and wrote the initial manuscript; B.X. participated in a portion of the experiments; K.B., D.C., K.X., and Y.Z. revised the manuscript. All authors approved the final version.

## Supporting information



Supporting Information

## Data Availability

The data that support the findings of this study are available on request from the corresponding author. The data are not publicly available due to privacy or ethical restrictions.
