# Supplementary material for: Punicalagin Enhances Autophagy Through Sirtuin 1/FoxO3a Axis to Inhibit Intracellular Mycobacterium Abscessus Infection
Source: Adv Sci (Weinh). 2025 Oct 14;13(2):e11734. doi: 10.1002/advs.202511734 (PMC12786361; doi:10.1002/advs.202511734)
Supplement: Supplementary file 1 — Supporting Information [file ADVS-13-e11734-s001.docx]

**Figure S1** UHPLC–MS analysis of ellagic acid in cell culture supernatant following co-culture of punicalagin with THP-1 cells


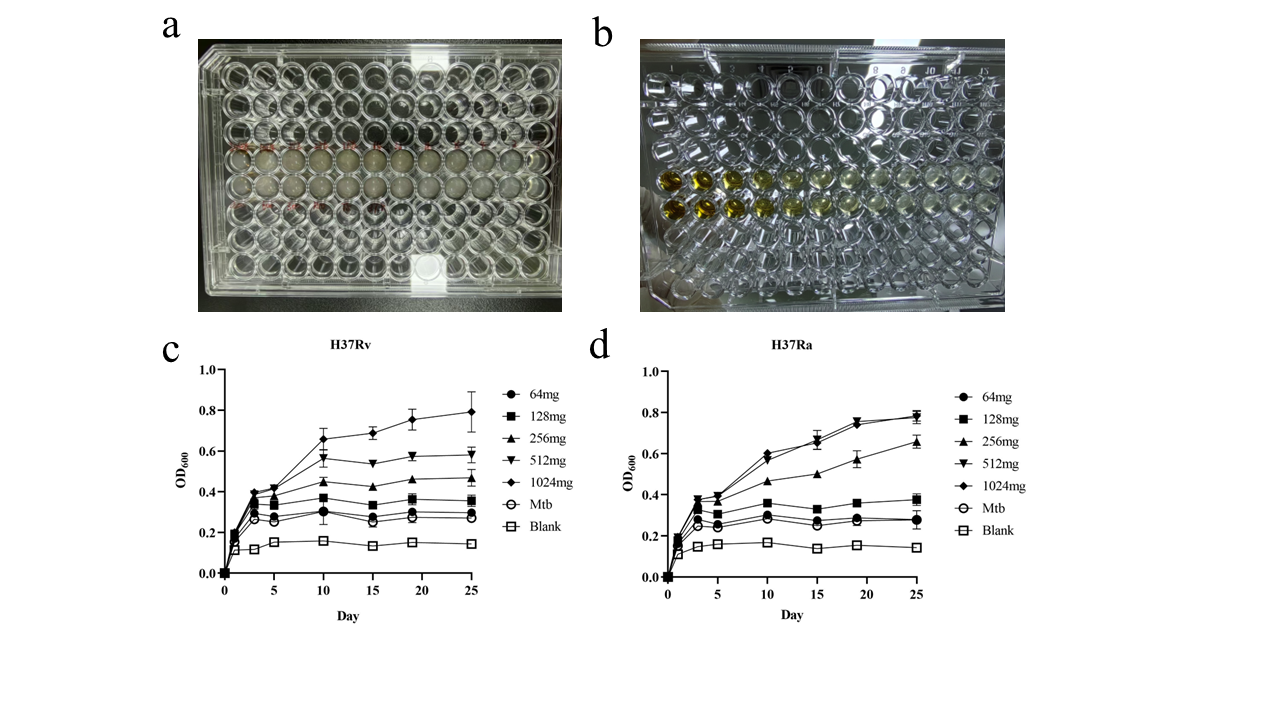


**Figure S2.** Punicalagin does not markedly inhibit mycobacterial growth in 7H9 medium culture. (a,b) Representative 96-well microdilution plate containing serial dilutions of punicalagin (1-2,048 μg mL⁻¹) prepared in Middlebrook 7H9 supplemented with 10% OADC and 0.05% Tween-80. (c) Growth of Mycobacterium tuberculosis H37Rv with punicalagin. (d) Growth of Mycobacterium tuberculosis H37Ra with punicalagin. “Control/*Mtb*” denotes vehicle-treated bacteria; “Blank” denotes medium only. Data are mean ± SEM of technical triplicates and are representative of ≥2 independent experiments.


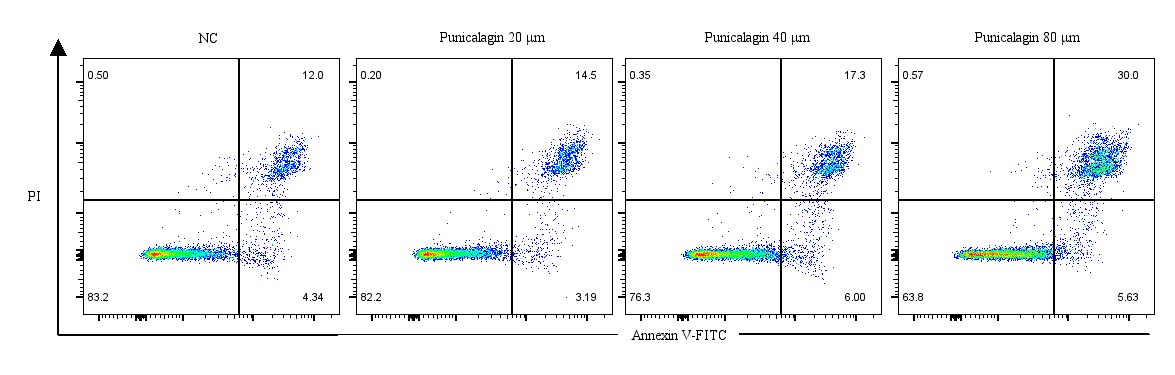


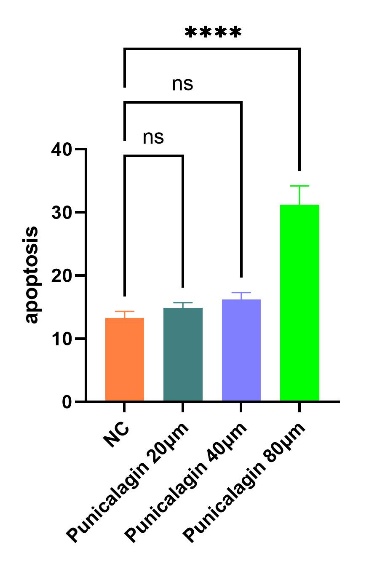


**Figure S3** Apoptosis levels and quantitative analysis of apoptosis were assessed after 24 hours of co-culture of punicalagin with THP-1 macrophages under uninfected conditions.

**Figure S4. Punicalagin limits MAB burden and apoptosis through a SIRT1-dependent autophagy program, whereas ellagic acid shows only partial effects.**
(a) Annexin V-FITC and PI staining of uninfected THP-1 macrophages treated with ellagic acid for 24 h. (mean ± SEM; ***, p<0.001; ****, p<0.0001 versus control). (b) Intracellular MAB measured as GFP fluorescence after infection with GFP-MAB for 4h and treatment with punicalagin (40 μM) or ellagic acid (40 or 80 μM) in 24h. (mean ± SEM; *, p<0.05; ns, not significant). (c) Annexin V/PI analysis in MAB-infected cells, treat with punicalagin (40 μM) or ellagic acid (40 or 80 μM). (mean ± SEM; *, p<0.05; **, p<0.01 versus MAB).

**Figure S5 Punicalagin treatment remodels the proteome of MAB-infected THP-1 macrophages.** (a) PCA of the proteomes from MAB-infected (M) and punicalagin-treated (P) THP-1 macrophages. (b) Number of up-regulated and down-regulated proteins in the punicalagin-treated group compared to the control. (c) Heatmap of representative differentially expressed proteins (DEPs).

**Figure S6 Molecular docking of punicalagin to host defense proteins implicated in macrophage control of MAB.** Zoomed panels highlight key contacting residues (blue) and the rightmost tables report hydrogen bonds and hydrophobic contacts extracted with PLIP (distances in Å). Protein structures were retrieved from the RCSB PDB. (a) Macrophage migration inhibitory factor (MIF) trimer (PDB 4GUM) (b) TIR domain of MyD88 (PDB 4DOM) (c) RP105/MD-1 complex (PDB 3B2D) (d) PTK2/FAK (PDB 2JKK). (e) TLR1-TLR2 heterodimer (PDB 2Z7X)

| Gene | Primer sequences（5’→ 3’） |
| --- | --- |
| *ActB* | GTCACCAACTGGGACGACAT  GTACATGGCTGGGGTGTTGA |
| *Atg7* | CAGTTTGCCCCTTTTAGTAGTGC  CCAGCCGATACTCGTTCAGC |
| *Atg14* | GCGCCAAATGCGTTCAGAG  AGTCGGCTTAACCTTTCCTTCT |
| *Nrf2* | TCAGCGACGGAAAGAGTATGA  CCACTGGTTTCTGACTGGATGT |
| *PIK3* | TATTTGGACTTTGCGACAAGACT  TCGAACGTACTGGTCTGGATAG |
| *PDK1* | CTGTGATACGGATCAGAAACCG  TCCACCAAACAATAAAGAGTGCT |
| *Sirt1* | TAGCCTTGTCAGATAAGGAAGGA  ACAGCTTCACAGTCAACTTTGT |

**Table 1** The primer sequences
